# Modulation of PKCα/ETS1 by klotho restores CYB5R4-dependent mitochondrial function in proximal tubular epithelial cells to attenuate the progression of diabetic kidney disease

**DOI:** 10.1186/s12933-026-03150-y

**Published:** 2026-03-28

**Authors:** Chun Gan, Xindi Zhou, Lan Qiu, Dan Chen, Yulu Shi, Qing Yang, Huimin Jiang, Han Xiao, Wanbing Chen, Xuejun Yang, Yaxi Chen, Mo Wang, Haiping Yang, Wei Jiang, Qiu Li

**Affiliations:** 1 Department of Nephrology, Children’s Hospital of Chongqing Medical University, National Clinical Research Center for Children and Adolescents’ Health and Diseases, Ministry of Education Key Laboratory of Child Development and Disorders, Chongqing Key Laboratory of Pediatric Metabolism and Inflammatory Diseases, Chongqing, People’s Republic of China; 2 Department of Pediatric Renal Rheumatology, Affiliated Hospital of Guizhou Medical University, Guizhou Provincial Children’s Medical Center, Guiyang, Guizhou People’s Republic of China; 3 Department of Pediatrics, Affiliated Hospital of North Sichuan Medical College, Nanchong, 637000 People’s Republic of China; 4 Centre for Lipid Research and Key Laboratory of Molecular Biology for Infectious Diseases (Ministry of Education), Institute for Viral Hepatitis, Department of Infectious Diseases, The Second Affiliated Hospital, Chongqing Medical University, Chongqing, People’s Republic of China

**Keywords:** Diabetic kidney disease, PTEC, ETS1, CYB5R4, Mitochondrial function

## Abstract

**Objective:**

Diabetic kidney disease (DKD) progression involves early proximal tubular injury, which precedes podocyte injury. The protective role of the protein Klotho in DKD is well-documented, but its impact on early tubular injury and mitochondrial dysfunction in proximal tubule epithelial cells (PTECs) remains underexplored. This study aimed to determine whether Klotho alleviates DKD by targeting mitochondrial dysfunction in PTECs and to uncover the molecular mechanisms involved.

**Methods:**

The role of Klotho was investigated using human kidney biopsies from patients at different DKD stages and a diabetic mouse model (induced by high-fat diet and streptozotocin). In vivo and in vitro techniques, including immunofluorescence, Western blot, transmission electron microscopy, and single-cell RNA sequencing, were used to assess tubular injury, mitochondrial integrity, and key protein interactions. The function of a newly identified protein, CYB5R4, was validated using knockdown and overexpression approaches in mouse models and human kidney (HK-2) cells.

**Results:**

Our results reveal a novel molecular pathway where Klotho alleviates early tubular injury in DKD by targeting the mitochondrial protein CYB5R4. We demonstrate that CYB5R4 is critically downregulated in DKD and that its restoration is both necessary and sufficient for Klotho's protective effect on mitochondrial function in PTECs. This regulation follows a defined signaling cascade where Klotho suppresses PKCα, which in turn inhibits the transcription factor ETS1. This inhibition of ETS1 leads to the de-repression of the CYB5R4 promoter, ultimately reducing tubular apoptosis and injury. This CYB5R4-dependent mechanism positions CYB5R4 as a key therapeutic target.

**Conclusion:**

Our findings uncover a novel Klotho/PKCα/ETS1/CYB5R4 signaling axis in PTECs that restores mitochondrial function and mitigates DKD progression, offering a promising therapeutic target for managing DKD.

**Supplementary Information:**

The online version contains supplementary material available at 10.1186/s12933-026-03150-y.

## Background

Diabetic kidney disease (DKD), a prevalent and severe complication of diabetes mellitus (DM), remains the leading cause of end-stage renal disease (ESRD) worldwide [1–4]. Despite substantial advances in diabetes management, effective therapies for DKD are still lacking, impeded by its complex and multifactorial pathogenesis. Early research predominantly focused on glomerular dysfunction, including hyperfiltration, basement membrane thickening and podocyte loss [5–7]. However, accumulating evidence now underscores the critical role of tubular injury, particularly involving proximal tubular epithelial cells (PTECs), which are increasingly recognized as critical factors in both the initiation and progression of DKD [8–11]. PTECs are highly susceptible to hyperglycemia-induced injury, potentially triggering a cascade of downstream pathological events [12, 13]. Given the pivotal role of PTECs dysfunction in DKD pathogenesis, therapeutic strategies targeting this compartment hold considerable promise [14, 15]. Notably, therapies targeting PTECs, such as sodium-glucose cotransporter-2 (SGLT2) inhibitors, have demonstrated efficacy in delaying DKD progression [16]. The growing recognition of PTECs injury as a key driver of renal dysfunction emphasizes the need to better understand tubular mechanisms involved in the development of DKD.

As metabolically active organs, the kidneys rely heavily on mitochondrial energy production to sustain their functions, making them particularly vulnerable to the vascular complications of diabetes [17]. Mitochondria, complex organelles found in all cells, are the primary source of energy production in the kidney under both physiological and diabetic conditions [17, 18]. Mitochondrial dysfunction has emerged as an early hallmark of diabetic tubulopathy, often preceding proteinuria and histological abnormalities. Impairments in mitochondrial biogenesis, dynamics and redox balance are especially detrimental to PTECs, which are densely packed with mitochondria due to their high energy demands and dependence on aerobic metabolism [18–20]. Therefore, restoring mitochondrial functional integrity in PTECs is essential for preserving tubular function and attenuating the progression of DKD. Nevertheless, the molecular mechanisms driving mitochondrial dysfunction in PTECs that contribute to DKD progression remain to be fully elucidated.

The α-Klotho protein, commonly known as Klotho, is an anti-aging protein primarily expressed in various tissues, particularly renal tubular epithelial cells. Klotho therapy has demonstrated marked improvement in renal function and reduced renal fibrosis [21, 22]. Beyond its anti-aging properties, Klotho also exerts renoprotective effects within the context of DKD [23–25]. Nevertheless, the underlying mechanisms through which Klotho alleviates mitochondrial dysfunction in PTECs during DKD progression remain incompletely understood.

The cytochrome-*b*_5_ reductase (CYB5R) family of flavoproteins is critical for maintaining cellular redox balance. This family comprises five members that facilitate the transfer of electrons from NADH, typically via an electron carrier such as cytochrome *b*_5_ (CYB5), to the final substrate [26, 27]. Among these, CYB5R4, also known as NCB5OR, holds particular significance. Loss of CYB5R4 has been associated with DM, leading to mitochondrial dysfunction, disrupted ion channel signaling, impaired iron homeostasis, and severely altered hepatic lipid metabolism alongside progressive loss of white adipose tissue [28, 29]. In this study, we have identified CYB5R4 as a novel Klotho-dependent mediator of mitochondrial integrity in PTECs. Our findings also reveal that Klotho can restore CYB5R4-dependent mitochondrial function in PTECs under high glucose conditions. Further molecular investigations show that ETS1, a transcriptional repressor of CYB5R4, is activated through phosphorylation at threonine 38 by PKCα, promoting its nuclear translocation. Notably, Klotho inhibits PKCα expression, reducing ETS1 phosphorylation and preventing its nuclear translocation. By modulating the PKCα/ETS1 pathway, Klotho presents a potential therapeutic strategy to restore CYB5R4-mediated mitochondrial function in PTECs and mitigate the progression of DKD.

## Materials and methods

Supplementary materials provide a comprehensive description of all experimental procedures not included in the main manuscript.

### Clinical data

This study enrolled patients with DM at the Affiliated Hospital of North Sichuan Medical College, with approval from its Ethics Committee (Ethics Approval No. 2025ER443-1) and with written informed consent obtained from all participants. Patients were in accordance with the 2024 American Diabetes Association guidelines. Participants were stratified into three groups based on their urinary albumin-to-creatinine ratio (UACR) levels: the DM group (UACR < 30 mg/g), the early DKD group (30 mg/g ≤ UACR ≤ 300 mg/g) and the clinical DKD group (UACR > 300 mg/g). Relevant clinical characteristics of the participants, collected during routine care and quantified on standardized laboratory platforms, are summarized in Table 1. Renal biopsy specimens were classified according to the Renal Pathology Society (RPS) classification system (DKD I-IV) [30]. Control kidney tissues were obtained from non-diabetic patients undergoing nephrectomy for renal tumors. The study protocol was approved by the Research Ethics Committee of the Children’s Hospital of Chongqing Medical University (Ethics Approval No. 2024234).

**Table 1:** Demographic and biochemical profiles of all enrolled subjects

	DM (UACR < 30 mg/g)	Early DKD (30 ≤ UACR < 300 mg/g)	Clinical DKD (UACR ≥ 300 mg/g)
Sex (M/F)	610 (337/273)	430 (242/188)	630(387/243)
Age (year)	60.92 ± 11.98	62.67 ± 13.23	64.59 ± 12.79
BMI (kg/m^2^)	24.47 ± 5.07	24.66 ± 4.34	24.99 ± 3.94
SBP (mm Hg)	130.90 ± 20.59	135.10 ± 23.02	147.50 ± 24.11^a^
DBP (mm Hg)	75.96 ± 14.59	76.93 ± 16.85	83.67 ± 14.59
HbA_1c_ (%)	8.04 ± 2.09	8.41 ± 2.62	8.65 ± 2.68
TG (mmol/L)	1.92 ± 1.89	1.92 ± 1.58	2.13 ± 1.85
LDL-C (mmol/L)	2.33 ± 0.96	2.50 ± 0.92	2.77 ± 1.20
HDL-C (mmol/L)	1.18 ± 0.42	1.10 ± 0.38	1.07 ± 0.40
Urinary Immunoglobulin G (mg/L)	5.86 ± 3.76	22.89 ± 21.63	295.70 ± 323.60^a^
Urinary Transferrin (mg/L)	2.04 ± 0.37	6.62 ± 4.91	105.80 ± 103.20^a^
Urinary α1-Microglobulin (mg/L)	25.21 ± 30.78	68.86 ± 70.95^a^	117.10 ± 132.90^a^

Results are expressed as mean ± SD

DM, diabetes mellitus; DKD, diabetic kidney disease; UACR, urinary albumin-to-creatinine ratio; BMI, body mass index; SBP, systolic blood pressure; DBP, diastolic blood pressure; HbA_1c_, glycated hemoglobin; TG, triglyceride; LDL-C, Iow-density lipoprotein-cholesterol; HDL-C, high-density lipoprotein-cholesterol

^a^*P* < 0.001

### Animals and treatment

Male C57BL/6 mice were obtained from Beijing HFK Biotechnology Co., Ltd. (Beijing, China). Genotyping of Klotho gene-overexpressing transgenic mice and Klotho gene-deficient mice was performed as previously described in our published study [23]. Mice were fed a high-fat diet (HFD, D12492, Research Diets) for 4 weeks. Subsequently, the mice were intraperitoneally injected with streptozotocin (STZ, 55 mg/kg, T1507, TargetMol) for 7 consecutive days. Two weeks after the final injection, diabetes was confirmed if fasting blood glucose levels exceeded 11.1 mM. Urine samples were collected every four weeks for analysis. The levels of urinary microalbumin, urinary α1-MG, urinary β2-MG and urinary NGAL were quantified by ELISA (JL20493, JL12300, JL20213, JL11556, JONLNBIO). To investigate the role of CYB5R4, adeno-associated virus (AAV) serotype VJ966 (PackGene Biotech) was delivered via renal injection. Mice were anesthetized, and a small flank incision was made to expose the left kidney. A total of 2.5 × 10^11^ GC was slowly injected into the renal cortex using a microliter syringe, carrying either the CYB5R4 overexpression or knockdown plasmid. The AAV vector alone served as the control. Kidney tissues were harvested for subsequent analysis. All animal procedures were approved by the Ethics Committee of the Children’s Hospital of Chongqing Medical University (IACUC approval No. CHCMU-IACUC20240628006).

### ATP content measurement

Cellular ATP levels were quantified using an enhanced ATP assay kit (MA0440, MeilumBio). Briefly, HK-2 cells were fully lysed and collected for measurement, followed by adding ATP detection reagent. Luminescence was measured and final ATP levels were normalized to protein content and expressed as nmol/mg protein.

### Subcellular fractionation

Mitochondrial and cytosolic fractions were isolated from HK-2 cells using a commercial mitochondrial isolation kit (MP-007, Invent Biotechnologies) following the manufacturer's protocol. Briefly, cells were harvested, washed with ice-cold PBS, and lysed in Buffer A. The lysates were centrifuged to remove nuclei and unbroken cells. The resulting supernatant was supplemented with Buffer B and subjected to high-speed centrifugation to isolate the mitochondrial fraction. The mitochondrial pellet was washed and further purified by additional PBS centrifugation steps. The final mitochondrial pellet was solubilized in lysis buffer containing detergent for Western blot analysis. The supernatant from the initial high-speed centrifugation step was retained as the cytosolic fraction.

### Luciferase reporter assay

The *CYB5R4* promoter sequences, containing either the wild-type (WT) or mutant (Mut) ETS1 binding sites, were amplified using a high-fidelity DNA polymerase (AG12202, Accurate Biology) and purified from agarose gels. The resulting full-length WT and Mut CYB5R4 promoter fragments were then ligated into the pGL3-Basic luciferase reporter vector (Geecreate, Wuhan, China) using the ClonExpress II One Step Cloning Kit (C112, Vazyme). Each reporter construct was co-transfected into cells with or without an ETS1 overexpression plasmid. Forty-eight hours post-transfection, dual-luciferase activity was measured using the Dual-Luciferase Reporter Assay Kit (11402ES60, Yeasen). Relative promoter activity was determined by normalizing the firefly luciferase luminescence to that of Renilla luciferase (LUC/REN).

### Co-immunoprecipitation (Co-IP)

HK-2 cells were transiently transfected with Flag-tagged PKCα plasmid or an empty vector control. Forty-eight hours post-transfection, cells were harvested and lysed using IP lysis buffer (P0013, Beyotime) supplemented with 1% protease inhibitor cocktail. The lysates were incubated with Anti-Flag Magnetic Beads (B26102, Bimake) overnight at 4 °C. After incubation, the beads were washed thoroughly with lysis buffer to remove non-specific binding. Bound proteins were then eluted by boiling the beads in 2 × SDS loading buffer. The resulting immunoprecipitates, along with the whole cell lysates (input), were analyzed by Western blotting using the specified antibodies.

### Statistical analysis

Data are presented as mean ± SD from at least three independent experiments. Differences between two groups were analyzed using a t-test, while comparisons among multiple groups were performed using one-way ANOVA. A *p*-value of less than 0.05 was considered statistically significant. All statistical analyses were conducted using GraphPad Prism 10.

## Results

### Proximal tubular injury is an early event in the progression of DKD

**Fig. 1:**
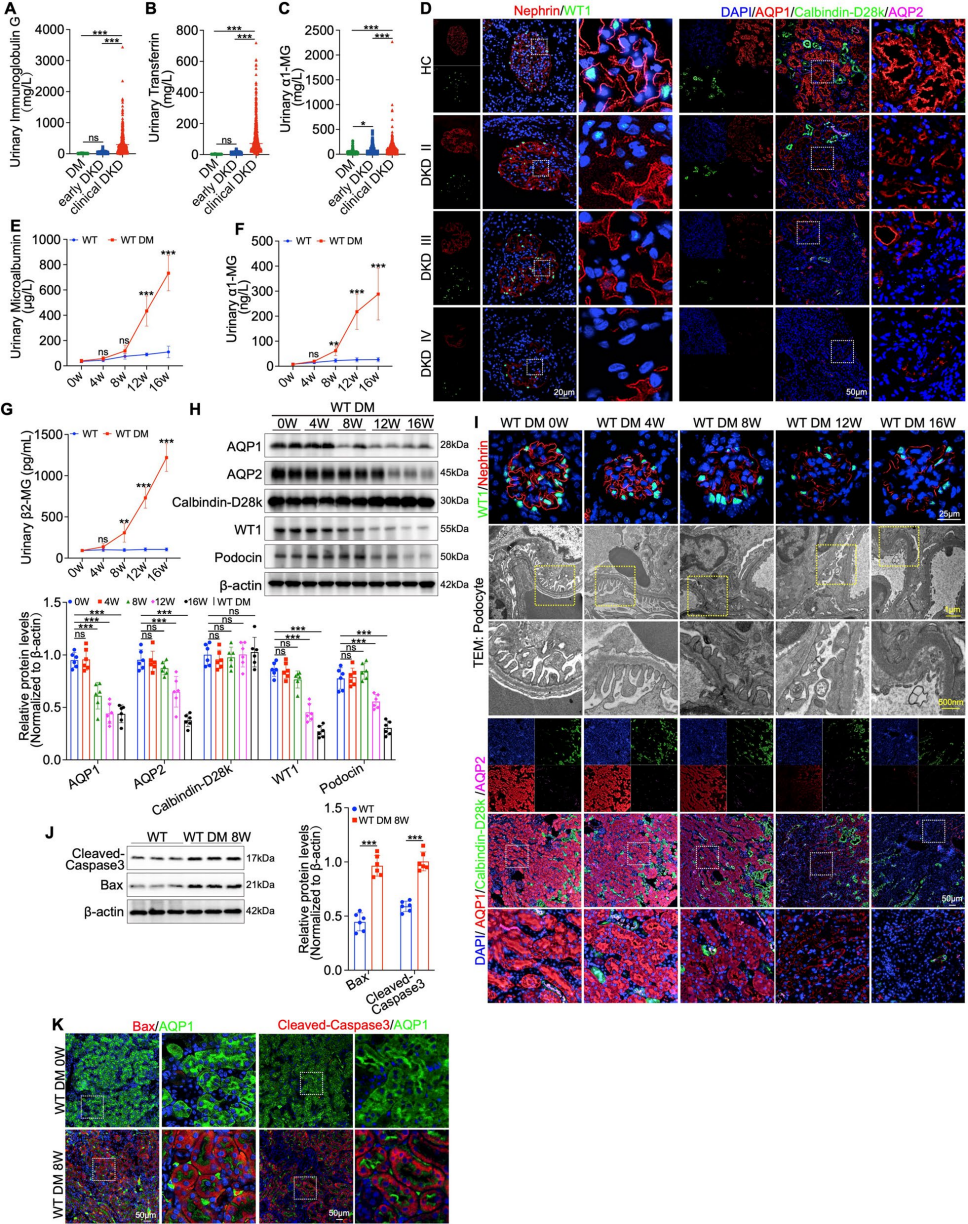
Proximal tubular injury precedes podocyte injury during the progression of DKD. **A**–**C** Comparison of urinary immunoglobulin G, urinary transferrin and urinary α1-MG levels among DM (n = 610), early DKD (n = 430), and clinical DKD (n = 630) patients. **D** IF staining showing colocalization of podocyte markers Nephrin, WT1, with proximal tubules marker AQP1, distal tubules marker Calbindin-D28k and the collecting ducts marker AQP2 among health control (HC) and different stage of DKD (II-IV) patients. **E**–**G** Longitudinal measurement of urinary microalbumin, urinary α1-MG, and urinary β2-MG in mouse groups over weeks (n = 6 per group). **H** Western blot analysis of AQP1, AQP2, Calbindin-D28k, WT1 and Podocin expression in diabetic mouse models as DM progresses (n = 6). **I** IF staining was used to assess the expression and localization of podocyte markers Nephrin and WT1, alongside tubular proteins AQP1, Calbindin-D28k and AQP2. TEM was utilized to evaluate ultrastructural changes in glomeruli. **J** Western blot analysis of apoptosis marker Cleaved-Caspase3 and Bax at 8 weeks post-model induction in diabetic mouse groups (n = 6). **K** IF staining showing colocalization of Bax and Cleaved-Caspase3 with AQP1 at week 8 post-modeling. Data are expressed as mean ± SD. Statistical significance is indicated as *ns*, no significant, **P < 0.01, ***P < 0.001

To investigate the role of proximal tubular injury in DKD progression, we first measured urinary markers of glomerular filtration barrier injury (IgG and transferrin) and tubular dysfunction (α1-microglobulin, α1-MG) in patients with DM, early-stage DKD (early DKD) and those with advanced DKD (clinical DKD) (Table 1) [31, 32]. Compared to the DM group, no significant increase in urinary IgG or transferrin was observed in early DKD (Fig. 1A, B). In contrast, urinary α1-MG levels were significantly elevated in the early DKD group compared to the DM group, remaining high in those with clinical DKD (Fig. 1C). Histological analysis of renal biopsies across different DKD stages (H&E and PAS staining) revealed characteristic pathological changes, including mesangial matrix expansion and glomerular basement membrane thickening, along with tubular vacuolar degeneration, along with their demographic and biochemical profiles (Figure S1A–C and Table 2). Immunofluorescence analysis showed that, compared to podocyte injury, characterized by disruption of linear Nephrin expression on the membrane and diminished expression of the nuclear marker WT1, tubular injury was markedly more pronounced in the early stages of DKD. This was particularly evident in the proximal tubules, as indicated by AQP1 labeling and further confirmed by the upregulated expression of NGAL, compared to the distal tubules denoted by Calbindin-D28k and the collecting ducts marked by AQP2 (Fig. 1D and Figure S1A).

**Table 2:** Demographic and biochemical profiles of renal biopsy patients

	DKD II	DKD III	DKD IV
Sex (M/F)	6 (4/2)	6 (3/3)	6(3/3)
Age (year)	61.33 ± 11.29	61.17 ± 8.89	61.13 ± 14.38
BMI (kg/m^2^)	24.82 ± 3.00	24.27 ± 4.53	25.05 ± 3.93
SBP (mm Hg)	130.50 ± 16.65	135.80 ± 16.07	158.50 ± 25.15
DBP (mm Hg)	86.00 ± 13.89	86.23 ± 8.15	87.83 ± 17.27
HbA_1c_ (%)	7.96 ± 1.61	8.12 ± 1.69	8.44 ± 2.16
TG (mmol/L)	1.74 ± 0.95	1.78 ± 1.23	1.79 ± 0.90
LDL-C (mmol/L)	2.13 ± 1.11	2.98 ± 0.91	2.92 ± 0.82
HDL-C (mmol/L)	1.28 ± 0.34	1.25 ± 0.42	1.01 ± 0.25
Urinary Immunoglobulin G (mg/L)	105.00 ± 102.40	440.60 ± 940.90	881.40 ± 1292.00
Urinary Transferrin (mg/L)	45.26 ± 40.73	146.50 ± 299.10	184.10 ± 207.10
Urinary α1-Microglobulin (mg/L)	45.29 ± 71.36	60.28 ± 63.49	154.5 ± 36.65^a^

Results are expressed as mean ± SD

BMI, body mass index; SBP, systolic blood pressure; DBP, diastolic blood pressure; HbA_1c_, glycated hemoglobin; TG, triglyceride; LDL-C, Iow-density lipoprotein-cholesterol; HDL-C, high-density lipoprotein-cholesterol

^a^*P* < 0.05

To validate these findings in vivo, we induced diabetes in mice using a high-fat diet (HFD) and streptozotocin (STZ) (Figure S1D). Urinary levels of microalbumin, α1-MG, β2-MG and NGAL were measured at weeks 0, 4, 8, 12 and 16 post-modeling. Levels of α1-MG, β2-MG and NGAL increased significantly by week 8, preceding a substantial increase in microalbumin that reached significance after week 12 (Fig. 1E-G, and Figure S1E). HE and PAS staining indicated that during the progression of DM, tubular vacuolar degeneration occurred prior to glomerular pathological changes. Immunofluorescence further revealed a concomitant reduction of LTL (a brush border glycoprotein) with an upregulation of SGLT2 and NGAL in proximal tubules (Figure S1F, G). Western blot analysis of kidney tissue from diabetic mice revealed dynamic changes in the expression of tubular and podocyte markers. Specifically, AQP1 expression in the proximal tubules was significantly downregulated as early as week 8, indicating initial tubular injury (Fig. 1H). Further immunofluorescence and transmission electron microscopy confirmed that podocyte injury, characterized by abnormal Nephrin/WT1 expression and foot process fusion, did not manifest until week 12 post-modeling, corroborated by Synaptopodin immunohistochemistry (Fig. 1I and Figure S1H). In contrast, increased apoptosis, evidenced by elevated Bax and Caspase-3 levels, also further demonstrated by the co-localization of Bax and Cleaved-Caspase3 within proximal tubules, was evident by week 8, suggesting that proximal tubular injury may precede podocyte dysfunction (Fig. 1J, K). These findings suggest that proximal tubular injury is an early event in DKD progression, occurring prior to podocyte injury.

### Klotho could alleviate mitochondrial dysfunction in PTECs to attenuate DKD progression

**Fig. 2:**
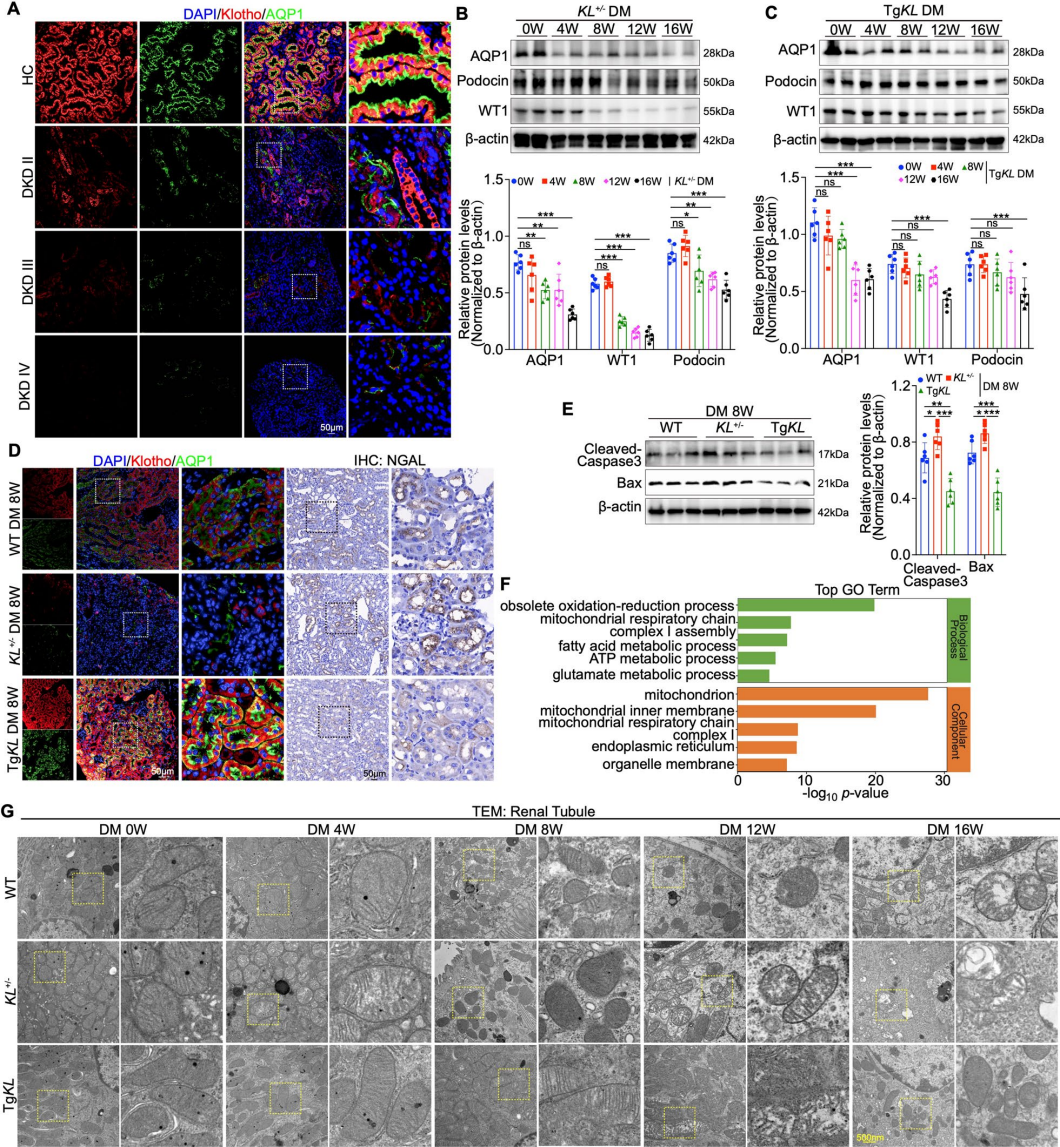
Klotho attenuates DKD progression by protecting PTECs function. **A** IF staining showing colocalization of Klotho with AQP1 in HC and patients with DKD (II-IV). **B**, **C** Western blot analysis of AQP1, WT1 and Podocin expressions in diabetic mouse models over several weeks (n = 6). **D** IF for Klotho with AQP1 co-localization and IHC for NGAL expression in kidney sections at week 8 post-modeling. **E** Western blot analysis of Cleaved-Caspase3 and Bax at week 8 post-modeling (n = 6). **F** Significantly enriched GO terms for gene differentially expressed in proximal tubule. **G** Representative TEM images of PTEC mitochondria. Data are expressed as mean ± SD. Statistical significance is indicated as *ns*, no significant, *P < 0.05, **P < 0.01, ***P < 0.001

Our previous studies demonstrated that Klotho primarily targets the glomerulus to delay DKD progression [23, 25]. However, its role in the early event of proximal tubular injury during DKD progression remains unclear. To address this, we analyzed kidney biopsy samples from DKD patients at different clinical stages, as well as kidney tissue samples from diabetic mice at the aforementioned time points, assessing the relationship between Klotho expression and proximal tubular injury using immunofluorescence staining. Results showed a progressive decline in both Klotho and AQP1 levels with increasing DKD severity (Fig. 2A and Figure S2A). In diabetic mice with Klotho deficiency or overexpression, Klotho conferred protection to proximal tubules as early as week 8, delaying subsequent podocyte injury. This was evidenced by reduced tubular vacuolar degeneration, reduction of urinary NGAL, attenuation of glomerular mesangial expansion and basement membrane thickening, maintenance of podocyte cytoskeleton and preservation of podocyte foot processes (Figure S2B–F). Preservation of podocyte markers, including Podocin, Nephrin, and WT1, was confirmed through Western blot and immunofluorescence analyses (Fig. 2B, C and Figure S3A). The protective effect of Klotho on proximal tubules at week 8 was further validated through immunofluorescence and Western blot assays (Fig. 2D, E).

To explore the molecular mechanisms driving proximal tubular injury during the early progression of DKD, we analyzed kidney subcellular expression profiles. Using publicly available single-cell RNA sequencing (scRNA-seq) data from diabetic mice (Gene Expression Omnibus, GEO Accession Number: GSE261356), we focused on PTECs exhibiting pronounced alterations (Figure S3B). Gene Ontology (GO) enrichment analysis of this cell subset indicated notable enrichment in energy metabolism-related pathways, with cellular components predominantly localized to the mitochondria (Fig. 2F). These findings suggest that, under diabetic conditions, PTECs may undergo substantial functional changes primarily characterized by dysregulated mitochondrial energy metabolism. Guided by these observations, we used transmission electron microscopy to examine mitochondrial structure in PTECs and found that by week 8 of DM progression, Klotho treatment preserved mitochondrial morphology while markedly reducing cristae disruption, a finding consistently supported by in vitro experiments wherein Klotho restored mitochondrial morphology and reduced fragmentation in high glucose-treated HK-2 cells, evidenced by transmission electron microscopy and Mitotracker staining (Fig. 2G and Figure S3C).

**Fig. 3:**
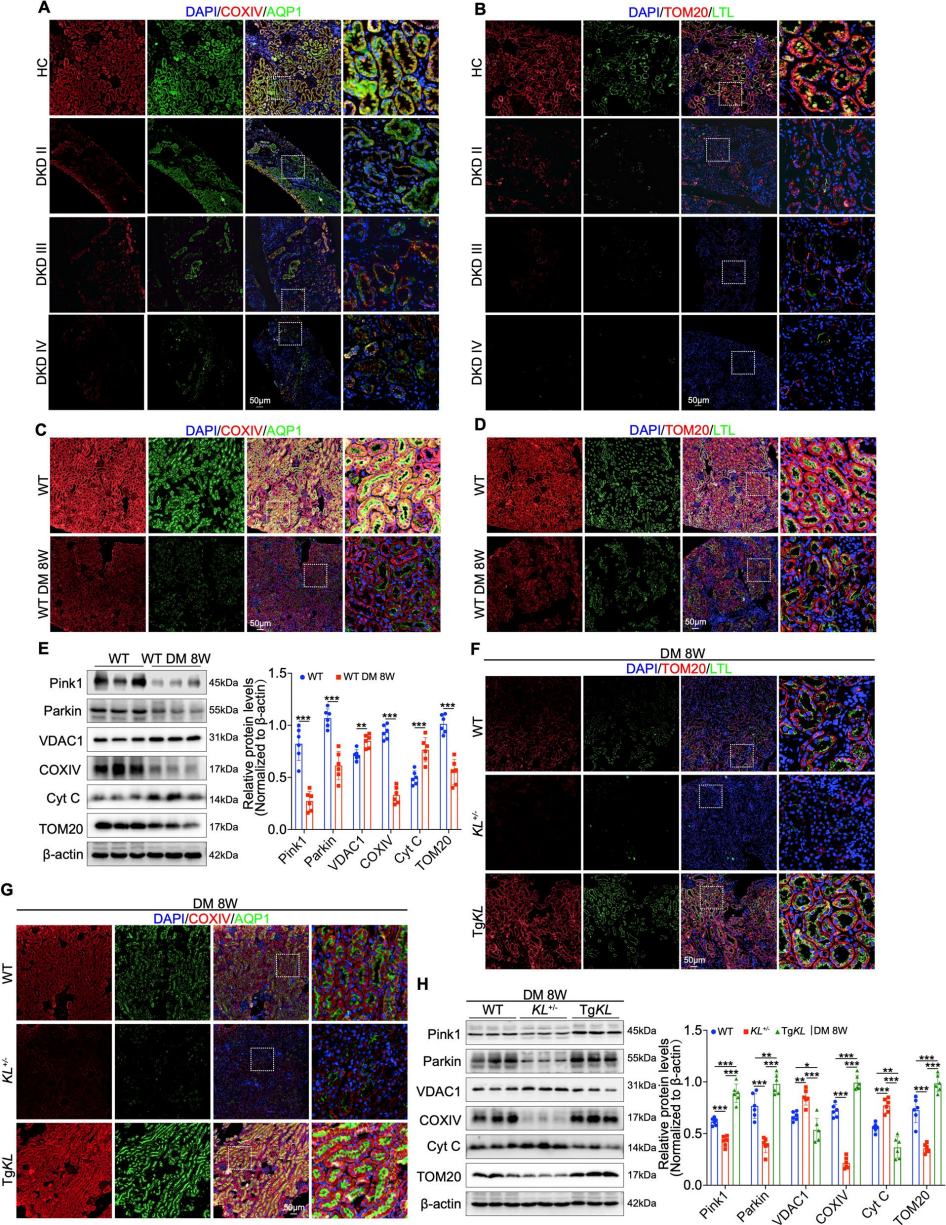
Klotho could restore mitochondrial function in PTECs. **A**, **B** IF staining showing colocalization of mitochondrial markers COXIV, TOM20 with PTECs markers AQP1, LTL in HC and DKD (stages II-IV). **C**, **D** IF staining showing colocalization of COXIV, TOM20 with AQP1, LTL at week 8 post-modeling. **E** Western blot analysis of mitophagy markers Pink1, Parkin, mitochondria-mediated apoptosis markers VDAC1, Cytochrome C (Cyt C), and mitochondria markers COXIV, TOM20 at 8 weeks in mouse groups (n = 6). **F**, **G** IF staining showing colocalization of TOM20, COXIV with LTL, AQP1 at week 8 post-modeling. **H** Western blot analysis of Pink1, Parkin, VDAC1, Cyt C, COXIV and TOM20 at week 8 post-modeling (n = 6). Data are expressed as mean ± SD. Statistical significance is indicated as *P < 0.05, **P < 0.01, ***P < 0.001

To further evaluate mitochondrial integrity, we conducted double immunofluorescence staining on clinical DKD renal biopsy samples using COXIV and TOM20 as markers for the mitochondrial inner and outer membranes, respectively, alongside AQP1 and LTL as proximal tubule markers [33, 34]. The staining revealed a gradual decrease in COXIV and TOM20 fluorescence intensity within AQP1/LTL-positive PTECs as DKD progressed (Fig. 3A, B). This trend suggests a potential decline in mitochondrial integrity, implying that mitochondrial alterations in PTECs may occur early during the development of DKD (Fig. 3A, B). Consistently, diabetic mice exhibited mitochondrial abnormalities in PTECs by week 8 (Fig. 3C, D). Western blot analysis at week 8 indicated downregulation of mitochondrial quality control-related proteins Pink1, Parkin, COXIV and TOM20, accompanied by upregulation of VDAC1 and cytochrome c (Cyt C) [33, 35] (Fig. 3E). To determine whether Klotho modulates mitochondrial function in PTECs, we conducted double immunofluorescence staining at week 8 in diabetic mice using the same mitochondrial and proximal tubule markers, revealing that Klotho considerably improved mitochondrial integrity in PTECs (Fig. 3F, G). Western blot analysis confirmed that Klotho restored Pink1, Parkin, COXIV and TOM20 expression while reducing VDAC1 and Cyt C levels. In *vitro* experiments further showed that Klotho inhibited high glucose-induced apoptosis proteins Cleaved-Caspase3 and Bax, restored COXIV and TOM20 expression, and decreased VDAC1 while inhibiting Cyt C translocation to the cytosol in HK-2 cells (Fig. 3H and Figure S3D, E). Additionally, Klotho rescued the impaired cellular ATP levels, restored TOM20 expression, suppressed mitochondrial reactive oxygen species (ROS) production as indicated by MitoSOX staining, and stabilized mitochondrial membrane potential as measured by TMRE and JC-1 staining (Figure S3F–H). Collectively, these results indicate that Klotho alleviates mitochondrial dysfunction in PTECs, contributing to the attenuation of DKD progression.

### CYB5R4 mediates the Klotho-induced restoration of mitochondrial function

**Fig. 4:**
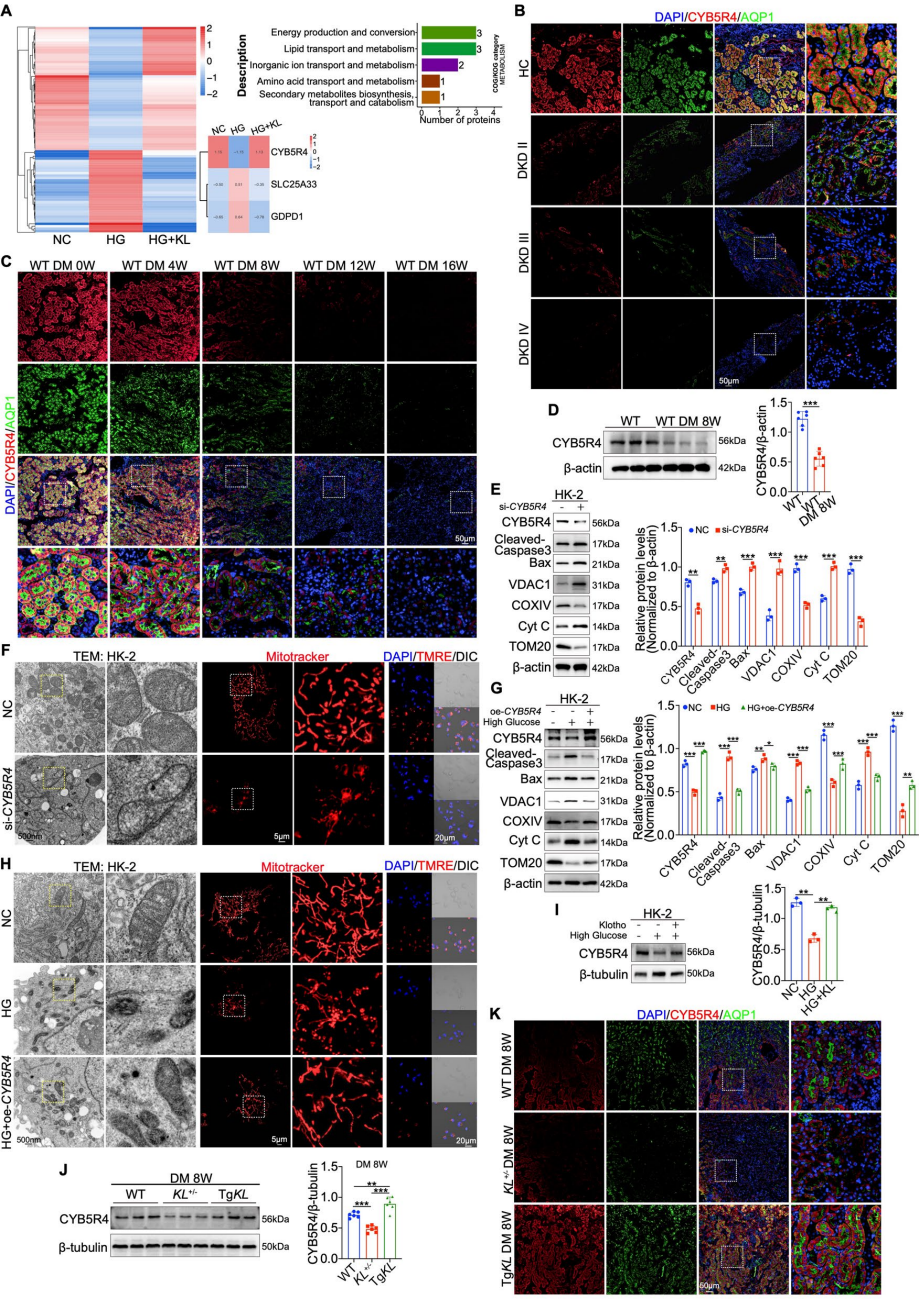
CYB5R4 mediates the restorative effects of Klotho on mitochondrial function in PTECs. **A** Heatmap and COG/KOG functional classification analysis in metabolism of differentially expressed proteins between HK-2 cells treated as high glucose (HG) and Klotho. **B**, **C** IF staining showing colocalization of CYB5R4 with AQP1 in DKD (II-IV) patients and mouse groups over weeks. **D** Western blot analysis of CYB5R4 at 8 weeks post-modeling (n = 6). **E**,**G** Western blot and quantitative analysis of CYB5R4, Cleaved-Caspase3, Bax, VDAC1, Cyt C, COXIV and TOM20 in CYB5R4 inhibition or overexpression (n = 3). **F**,**H** Representative images of PTECs’ mitochondria by TEM and Mitotracker, as well as assessment of mitochondrial membrane potential by TMRE in CYB5R4 inhibition or overexpression. **I** Western blot analysis of CYB5R4 in HK-2 cells treated with HG and Klotho (n = 3). **J** Western blot analysis of CYB5R4 at 8 weeks post-modeling (n = 6). **K** IF staining showing colocalization of CYB5R4 with AQP1 at 8 weeks post-modeling (n = 6). Data are expressed as mean ± SD. Statistical significance is indicated as *P < 0.05, **P < 0.01, ***P < 0.001

To identify Klotho-dependent mitochondrial mediators, we performed a quantitative proteomic analysis using 4D-FastDIA-based LC–MS/MS and identified CYB5R4, SLC25A33, and GDPD1 as candidate proteins involved in energy production and conversion (Fig. 4A). Among these candidates, CYB5R4 exhibited the most pronounced changes (Fig. 4A). Immunofluorescence analysis of DKD patient biopsies revealed a progressive decrease in CYB5R4 expression corresponding with disease severity, closely paralleling AQP1 expression (Fig. 4B). In diabetic mice, CYB5R4 was specifically expressed in PTECs and gradually declined as DM progressed to DKD, with the most significant reduction observed at week 8 post-modeling (Fig. 4C, D). In vitro, CYB5R4 knockdown in HK-2 cells triggered apoptosis, as shown by increased Cleaved-Caspase 3 and Bax levels, and resulted in mitochondrial dysfunction characterized by elevated VDAC1 and Cyt C expression, enhanced Cyt C translocation to the cytosol, and reduced COXIV and TOM20 expression (Fig. 4E and Figure S4A, B). Transmission electron microscopy and Mitotracker staining further confirmed enhanced mitochondrial fragmentation and ultrastructural disruption (Fig. 4F). CYB5R4 deficiency also led to a loss of mitochondrial membrane potential (TMRE and JC-1), decreased TOM20 expression, increased ROS production (MitoSOX) and reduced ATP production (Fig. 4F and Figure S4C, D, I). Comparable defects were observed in high glucose-treated HK-2 cells, while CYB5R4 overexpression effectively reversed these abnormalities (Fig. 4G, H and Figure S4E–I). Importantly, Western blotting and fluorescence analyses demonstrated that Klotho counteracted high glucose-induced suppression of CYB5R4 expression in HK-2 cells (Fig. 4I and Figure S4J). In *vivo*, CYB5R4 downregulation was exacerbated by Klotho deficiency but alleviated by Klotho overexpression at week 8 post-modeling, as confirmed by Western blot and immunofluorescence (Fig. 4J, K).

**Fig. 5:**
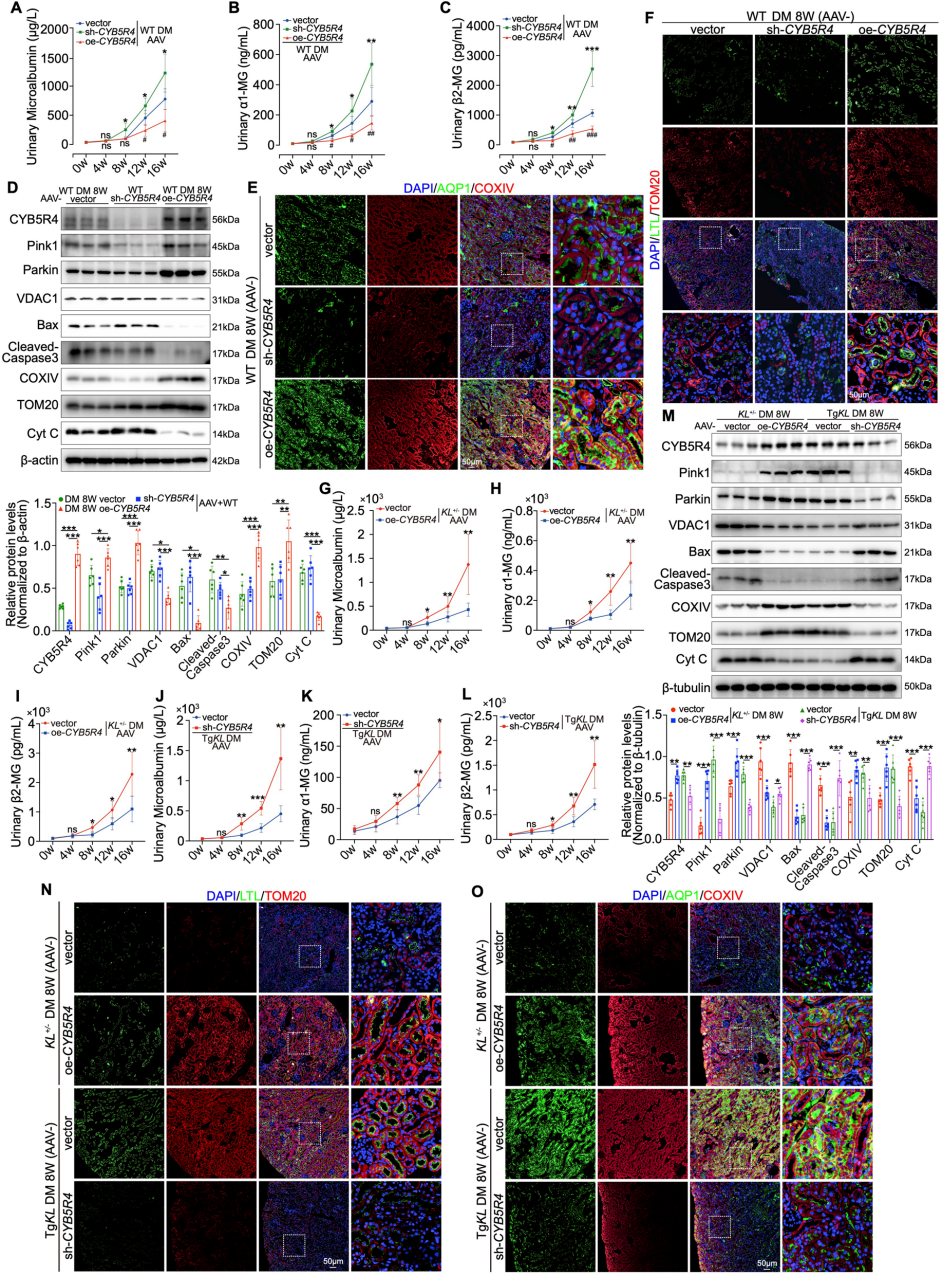
CYB5R4 is required for Klotho-induced renoprotection in diabetic mice. **A**–**C** Longitudinal measurements of urinary microalbumin, urinary α1-MG, and urinary β2-MG in diabetic mouse groups over several weeks (n = 6). **D** Western blot analysis of CYB5R4, Pink1, Parkin, VDAC1, Bax, Cleaved-Caspase3, COXIV, TOM20 and Cyt C in mouse groups (n = 6). **E**, **F** IF staining showing colocalization of COXIV, TOM20 with AQP1, LTL at 8 weeks post-modeling. **G**–**L** Longitudinal measurement of urinary microalbumin, urinary α1-MG, and urinary β2-MG in diabetic mouse groups over weeks (n = 6). **M** Western blot analysis of CYB5R4-mediated Klotho effects on the expression of CYB5R4, Pink1, Parkin, VDAC1, Bax, Cleaved-Caspase 3, COXIV, TOM20 and Cyt C at 8 weeks post-model induction (n = 6). **N**, **O** IF staining to further analyze the expression and localization of TOM20, COXIV, LTL and AQP1 at 8 weeks post-modeling. Data are expressed as mean ± SD. Statistical significance is indicated as *ns*, no significant, *P < 0.05, **P < 0.01, ***P < 0.001

Guided by this temporal pattern, we employed AAV-mediated renal in situ injection to modulate CYB5R4 expression in diabetic mice (Figure S5A). CYB5R4 knockdown accelerated and worsened glomerular filtration barrier injury, manifested by increased urinary microalbumin, mesangial expansion, and basement membrane thickening, along with aggravated tubular dysfunction indicated by elevated urinary α1-MG and β2-MG and tubular vacuolar degeneration. Conversely, CYB5R4 overexpression significantly ameliorated both glomerular and tubular injury (Fig. 5A–C and Figure S5B). At the mitochondrial level, CYB5R4 inhibition disrupted PTEC mitochondrial structure and suppressed the expression of Pink1, Parkin, COXIV, and TOM20, while upregulating VDAC1, Cyt C, Cleaved-Caspase 3, and Bax (Fig. 5D). Immunofluorescence further confirmed reduced COXIV and TOM20 colocalization with AQP1 and LTL, respectively, indicating impaired mitochondrial integrity. In contrast, CYB5R4 overexpression preserved mitochondrial morphology and protein expression (Fig. 5E, F). Moreover, AAV-mediated CYB5R4 overexpression in Klotho-deficient diabetic mice significantly rescued glomerular filtration barrier injury, as indicated by reduced urinary microalbumin, mesangial expansion and basement membrane thickening (Fig. 5G and Figure S5C). Concurrently, tubular injury was ameliorated, as evidenced by decreased urinary α1-MG and β2-MG, along with resolution of tubular vacuolar degeneration (Fig. 5H, I and Figure S5C). Conversely, in diabetic mice overexpressing Klotho, interference with CYB5R4 abolished Klotho’s protective effects, aggravating glomerular and tubular injury (Fig. 5J–L and Figure S5C). Furthermore, CYB5R4 overexpression restored mitochondrial homeostasis in PTECs from Klotho-deficient diabetic mice, as evidenced by recovery of Pink1, Parkin, COXIV, and TOM20 expression levels, alongside suppression of VDAC1, Cyt C, Cleaved-Caspase 3, and Bax in Western blot analyses. Immunofluorescence confirmed enhanced TOM20 and COXIV colocalization with LTL and AQP1, respectively. In contrast, CYB5R4 knockdown in Klotho-overexpressing mice reversed these protective effects (Fig. 5M–O).

Together, these findings identify CYB5R4 as a key mediator of Klotho’s ability to restore mitochondrial function in PTECs during DKD progression.

### CYB5R4 expression is inhibited by the transcription factor ETS1

**Fig. 6:**
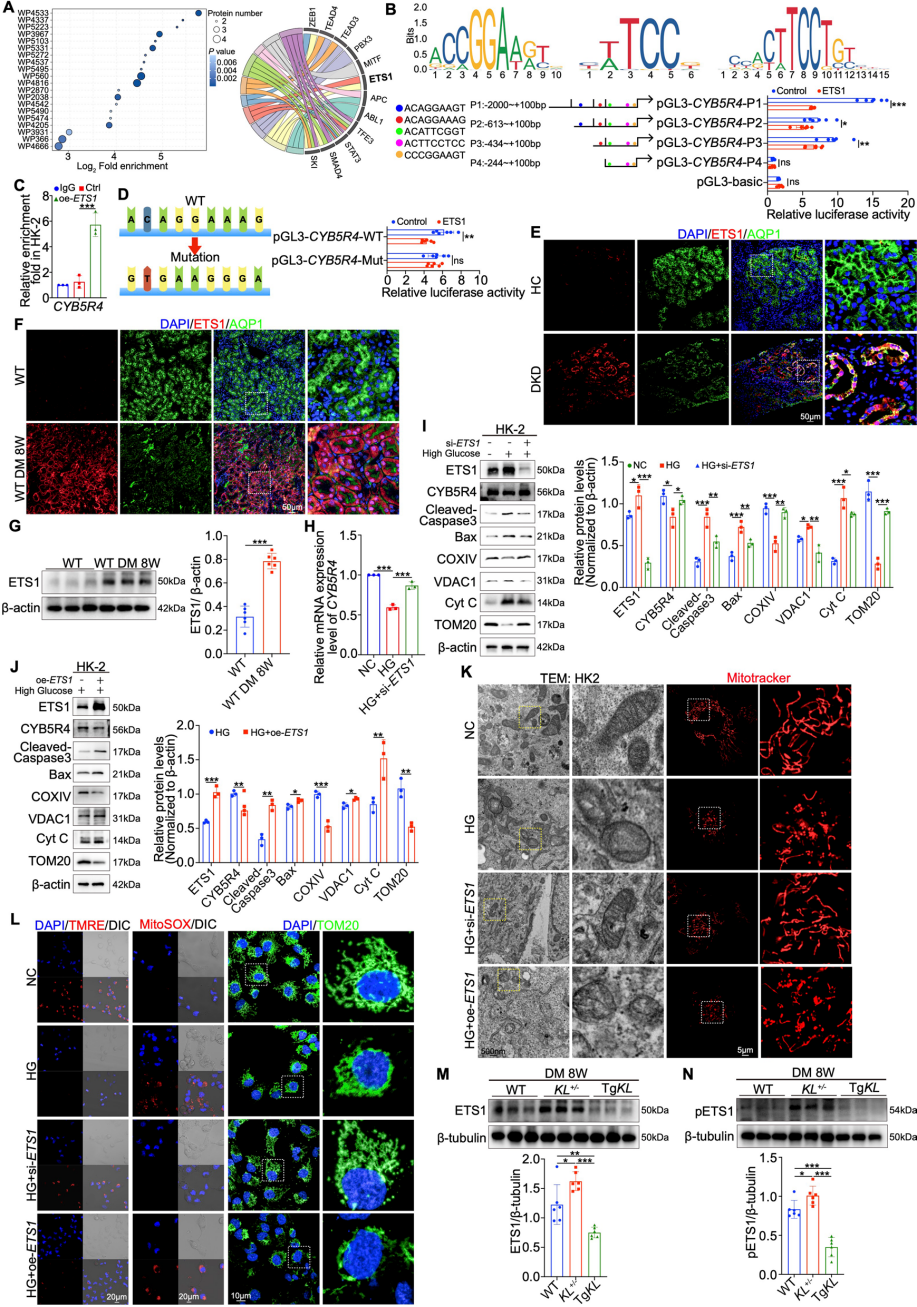
ETS1 transcriptionally represses CYB5R4 expression. **A** Identification of hub transcription factors within WikiPathways, visualized using dot plot and circos plot. **B**, **C** Prediction of ETS1 binding sites on the *CYB5R4* gene promoter using JASPAR, validated by dual-luciferase reporter assays and CUT&Tag-qPCR. **D** Luciferase activity of CYB5R4 binding promoter sequence (WT/mutation) in response to vector or ETS1 overexpression. **E**, **F** IF staining showing colocalization of ETS1 with AQP1 in DKD patients and DM mouse groups. **G** Western blot analysis of ETS1 at 8 weeks post-modeling (n = 6). **H** qPCR analysis of the effect of ETS1 inhibition on *CYB5R4* mRNA levels in high-glucose-induced HK-2 cells. **I**, **J** Western blot analysis of ETS1, CYB5R4, Cleaved-Caspase3, Bax, VDAC1, Cyt C, COXIV, TOM20 in ETS1 inhibition or overexpression conditions (n = 3). **K** Representative images of HK-2 cell mitochondria by TEM and Mitotracker in ETS1 inhibition or overexpression. **L** Assessment of HK-2 cell mitochondrial membrane potential by TMRE and mitochondrial superoxide levels by MitoSOX, as well as mitochondrial TOM20 morphology changes in ETS1 inhibition or overexpression. **M**, **N** Western blot analysis of ETS1, pETS1 at 8 weeks post-modeling (n = 6). Data are expressed as mean ± SD. Statistical significance is indicated as *ns*, no significant, *P < 0.05, **P < 0.01, ***P < 0.001

Having established the essential role of CYB5R4 in Klotho-mediated mitochondrial protective effects, we then explored its regulatory mechanism. As Klotho is not a transcription factor, we employed DNA pull-down combined with mass spectrometry to identify ETS1 as a potential transcriptional regulator (Fig. 6A and Figure S6A, B). JASPAR, a database of transcription factor binding profiles, predicted five ETS1 binding motifs within the *CYB5R4* gene promoter region (− 2000 to + 100 bp). Dual-luciferase reporter assays and CUT&Tag-qPCR demonstrated that ETS1 binds to a core site (ACAGGAAAG) within the -434 to -244 bp region, thereby suppressing *CYB5R4* transcription (Fig. 6B, C). Mutation of this core motif abolished the inhibitory effect, confirming that ETS1 functions as a transcriptional repressor of CYB5R4, with this binding site critical for its repressive activity (Fig. 6D). Immunofluorescence of human DKD biopsies revealed increased nuclear ETS1 in PTECs, a finding confirmed at week 8 post-modeling and validated through Western blotting confirmation (Fig. 6E–G). In HK-2 cells, ETS1 knockdown relieved high glucose-induced suppression of CYB5R4 transcription and expression, restoring COXIV and TOM20 expression while reducing VDAC1, Cyt C, Cleaved-Caspase 3, and Bax levels (Fig. 6H, I and Figure S6C). These changes were accompanied by improved mitochondrial morphology marked by reduced fragmentation (observed by transmission electron microscopy and MitoTracker staining), stabilization of mitochondrial membrane potential (TMRE and JC-1), decreased ROS production (MitoSOX), corroborated by TOM20 immunofluorescence (Fig. 6K, L and Figure S6D). Conversely, ETS1 overexpression elicited opposite effects (Fig. 6J–L and Figure S6C, D).

Further experiments showed that Klotho inhibited high glucose-induced ETS1 expression and nuclear accumulation in HK-2 cells, as well as suppressing ETS1 expression in vivo at week 8 post-modeling (Fig. 6M and Figure S6E, F). Given that ETS1 phosphorylation reflects its activated state [36], we then examined clinical DKD samples and found progressive nuclear accumulation of phosphorylated ETS1 in PTECs corresponding with disease severity, which Klotho prevented this aggregation in diabetic mice at week 8 post-modeling (Fig. 6N and Figure S6G, H). Collectively, these findings signify that Klotho preserves CYB5R4 expression by inhibiting ETS1 activation, thereby maintaining mitochondrial function in PTECs.

### PKCα downregulation by Klotho suppresses ETS1 activation

**Fig. 7:**
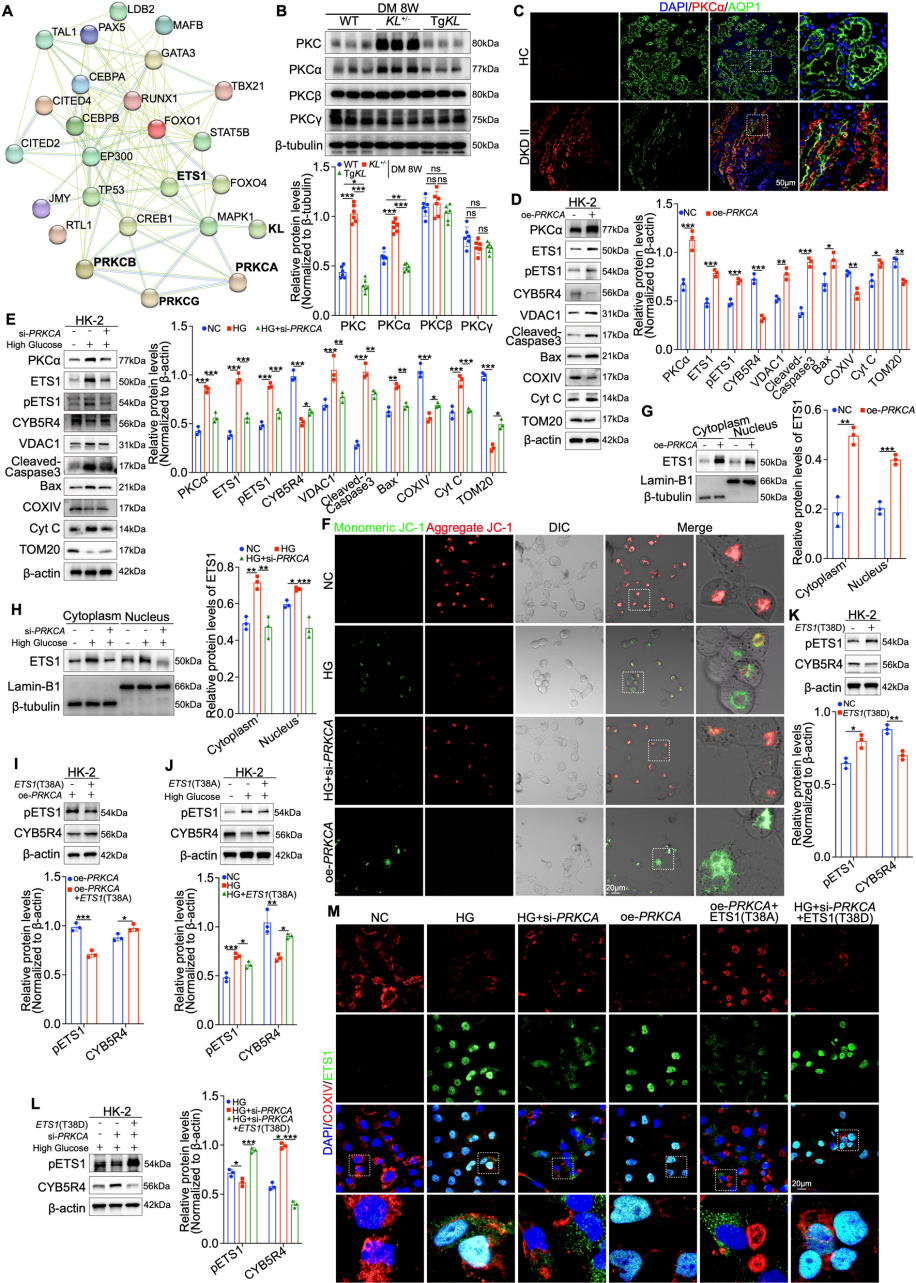
PKCα phosphorylates ETS1 to enhance its transcriptional activation. **A** Association of the PKC family protein with Klotho and ETS1, as identified in the STRING database. **B** Western blot analysis of PKC, classical PKC isoforms (α, β, and γ) at 8 weeks in diabetic mouse groups (n = 6). **C** IF staining showing colocalization of PKCα with AQP1 in DKD patients. **D**, **E** Western blot analysis of PKCα ETS1, pETS1, CYB5R4, VDAC1, Cleaved-Caspase3, Bax, COXIV, Cyt C and TOM20 in PKCα inhibition or overexpression (n = 3). **F** Assessment of mitochondrial membrane potential by JC-1 in PKCα inhibition or overexpression. **G**, **H** Western blot analysis of ETS1 subcellular localization in PKCα inhibition or overexpression. **I**, **J** Western blot analysis of pETS1 and CYB5R4 in conditions with reduced ETS1 phosphorylation (ETS1, T38A). **K**, **L** Western blot and quantitative analysis of pETS1 and CYB5R4 in conditions with increased ETS1 phosphorylation (ETS1, T38D). **M** IF staining showing localization of ETS1 with COXIV in the indicated groups. Data are expressed as mean ± SD. Statistical significance is indicated as *ns*, no significant, *P < 0.05, **P < 0.01, ***P < 0.001

Having confirmed that Klotho inhibits ETS1 activation by reducing its phosphorylation, we hypothesized the involvement of a specific kinase mediating this effect. To investigate this, we utilized STRING (functional protein association networks) to analyze potential interactions between Klotho and ETS1, focusing on protein kinases. Among the candidates, the PKC family was highlighted as particularly noteworthy, aligning with our previous findings linking Klotho to PKCα in podocytes [25] (Fig. 7A). We then assessed the expression of PKC isoforms (α, β, γ) in clinical DKD samples and diabetic mouse kidneys at week 8 post-modeling using immunofluorescence and immunohistochemistry. PKC expression was markedly elevated in PTECs during early DM (Figure S7A–D). Furthermore, Western blot, fluorescence and immunohistochemistry analyses demonstrated a negative correlation between PKC expression and Klotho levels (Fig. 7B and Figure S7E, F). Notably, PKCα exhibited the strongest correlation, with the increase in PKC expression under pathological conditions primarily driven by PKCα (Fig. 7B, C and Figure S7G, H). In vitro, we performed PKCα overexpression and knockdown in both HK-2 cells and high-glucose-treated HK-2 cells. Similar to the effects observed under high-glucose conditions, immunofluorescence analysis showed that PKCα overexpression suppressed CYB5R4 expression. This was further confirmed by Western blot results, demonstrating that PKCα overexpression simultaneously inhibited the expression of COXIV and TOM20, while increasing the levels of ETS1, p-ETS1, VDAC1, Cyt C, Cleaved Caspase-3, and Bax (Fig. 7D and Figure S8A). These molecular alterations were accompanied by improved mitochondrial morphology, stabilized mitochondrial membrane potential, and reduced ROS production, validated by TOM20 immunofluorescence (Fig. 7F and Figure S8A, B). In contrast, PKCα knockdown reversed the aforementioned high glucose-induced changes (Fig. 7E, F and Figure S8A, B). We also identified that PKCα overexpression enhanced ETS1 accumulation in both the cytoplasm and nucleus of HK-2 cells, whereas PKCα silencing reduced high glucose-induced ETS1 expression in both compartments (Fig. 7G, H). To establish a direct mechanistic link between PKCα and ETS1, we performed Co-IP assays in HK-2 cells overexpressing Flag-tagged PKCα. Immunoprecipitation with an anti-Flag antibody followed by Western blotting confirmed a direct physical interaction between ETS1 and PKCα (Figure S8C). To probe the phosphorylation mechanism further, we mutated a critical ETS1 phosphorylation site at threonine 38 to alanine (T38A) to block its phosphorylation and subsequently overexpressed this mutant in HK-2 cells. Under this condition, even with PKCα overexpression, ETS1 phosphorylation decreased, accompanied by elevated CYB5R4 expression and a marked reduction in ETS1 levels in both the cytoplasm and nucleus (Fig. 7I and Figure S8D). Similarly, in high glucose-treated HK-2 cells, the ETS1 (T38A) mutation reduced ETS1 phosphorylation, increased CYB5R4 expression, and diminished ETS1 levels in both the cytoplasm and nucleus (Fig. 7J and Figure S8E). Conversely, the overexpression of a phosphomimetic ETS1 mutant (threonine mutated to aspartic acid at position 38, T38D) in normal HK-2 cells enhanced ETS1 phosphorylation, increased ETS1 levels in both the cytoplasm and nucleus, and suppressed CYB5R4 expression (Fig. 7K and Figure S8F). Moreover, in high glucose-treated HK-2 cells, even with PKCα knockdown, ETS1 (T38D) overexpression maintained elevated ETS1 phosphorylation, increased ETS1 accumulation in both compartments, and decreased CYB5R4 expression (Fig. 7L and Figure S8G). Collectively, these findings indicate that elevated PKCα expression under high-glucose conditions directly promotes ETS1 phosphorylation and activation, facilitating its nuclear translocation. This conclusion is further supported by immunofluorescence analysis in HK-2 cells (Fig. 7M).

## Discussion

**Fig. 8:**
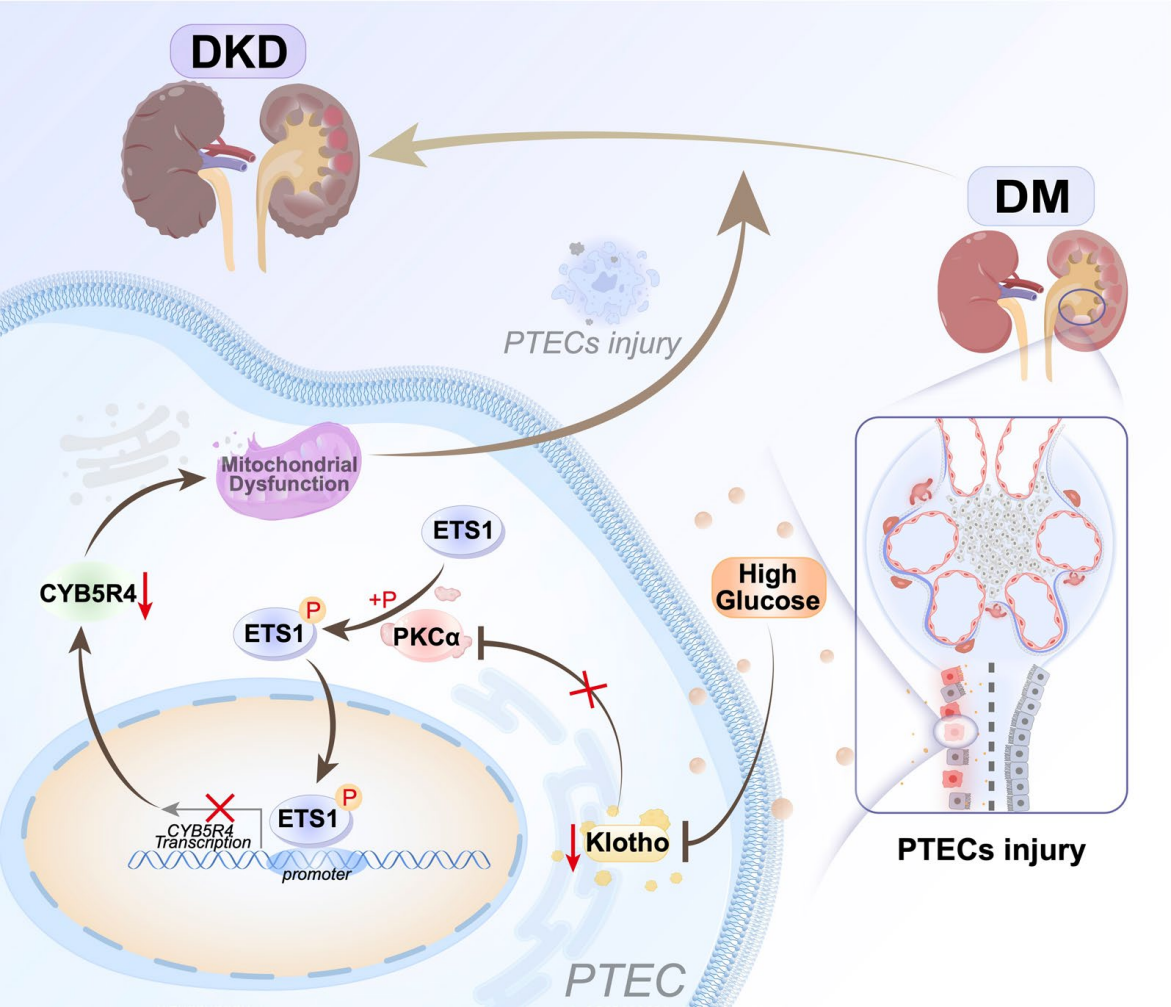
Overview of the study design showing how Klotho modulates the PKCα/ETS1 axis to restore CYB5R4-dependent mitochondrial function in PTECs, thereby attenuating the progression of DKD

DKD is a serious long-term complication of DM, accounting for nearly half of all chronic kidney disease cases, with approximately 40% of individuals with DM eventually developing DKD [18, 37]. The progression of DKD is highly variable, and its underlying mechanisms remain poorly elucidated. Recent evidence suggests a strong association between mitochondrial dysfunction and kidney disease. Emerging evidence indicates that mitochondrial dysfunction is a key contributor to kidney disease, with impaired renal mitochondria playing a central role in DKD pathogenesis [17–20, 38, 39]. Due to the kidneys’ high metabolic demands, mitochondrial energy production is essential for their function, rendering them particularly susceptible to the complications of diabetes. In PTECs, mitochondrial dysfunction, including defects in biogenesis, dynamics and redox balance, emerges as an early indicator of DKD. Restoration of mitochondrial function in PTECs is crucial for preserving kidney health; however, the molecular mechanisms driving mitochondrial dysfunction in DKD remain poorly understood. The present study provides novel insights into these mechanisms, demonstrating that the decline in Klotho expression in PTECs during the progression from DM to DKD initiates an increase in PKCα expression, which subsequently phosphorylates and activates ETS1. The activated ETS1 translocates to the nucleus, binds to the CYB5R4 gene promoter and suppresses CYB5R4 expression. This cascade of events contributes to mitochondrial dysfunction in PTECs, promoting the progression of DKD (Fig. 8).

Klotho exists as both a membrane-bound and soluble form, with the latter predominating in circulation and serving as a protective factor against tubular injury and a promising early biomarker for DKD [40, 41]. As a key regulator of phosphate and energy metabolism, Klotho also modulates cellular redox balance [42]. Its deficiency elevates ROS levels and aggravates oxidative stress, compromising mitochondrial function, while Klotho administration lowers ROS levels, mitigates oxidative stress and preserves mitochondrial integrity [43, 44]. However, the molecular mechanisms by which Klotho modulates mitochondrial function in PTECs to delay the progression from DM to DKD were previously unclear. In this study, we identify CYB5R4 as a Klotho-regulated mitochondrial protein in PTECs that contributes to protection against high glucose-induced mitochondrial dysfunction. While previous studies have primarily focused on CYB5R3, as mice with β-cell-specific deletion of CYB5R3 exhibit mitochondrial abnormalities, including a diminished respiratory response to glucose and defective secretory granules, leading to impaired insulin secretion and glucose intolerance [45]. Our findings demonstrate that CYB5R4 can similarly preserve mitochondrial function in PTECs and delay the onset and progression of DKD. The underlying mechanism may involve CYB5R4 deficiency under hyperglycemic conditions disrupting the connection between endoplasmic reticulum (ER) stress and mitochondrial dysfunction. This disruption impairs ER homeostasis, induces unresolved ER stress, and triggers apoptotic signaling and oxidative stress, ultimately leading to mitochondrial dysfunction [27, 28, 46]. Collectively, these mechanisms suggest that the protection of tubular mitochondria by Klotho/CYB5R4 may confer indirect glomerular protection, possibly by ameliorating tubuloglomerular feedback and improving the renal oxidative stress microenvironment to delay DKD progression. Furthermore, we reveal ETS1 as a transcriptional repressor of CYB5R4, showing that inhibition of ETS1 phosphorylation limits its nuclear translocation and restores CYB5R4 expression. Despite these advancements, the precise downstream mechanisms by which CYB5R4 regulates mitochondrial function warrant further investigation. To this end, we have generated PTEC-specific *CYB5R4* knockout mice to facilitate comprehensive future studies of CYB5R4-mediated mitochondrial energy metabolism. Beyond its role in regulating CYB5R4, ETS1 also contributes to renal and vascular injury. Knockdown of ETS1 reduces PTEC apoptosis and mitigates renal interstitial fibrosis via NLRP3 transcriptional regulation, thereby alleviating AKI [47–50]. These findings suggest that ETS1 may mediate tubular inflammatory responses in response to changes in Klotho expression, thereby participating in the progression from DM to DKD.

Finally, our study confirms that PKCα can enhance the phosphorylation of ETS1. ETS1 serves as an effector for PKCα to fulfil certain functions in cancers, including breast cancer [51, 52]. In addition to PKCα, other kinases, such as glycogen synthase kinase 3 beta and Src family kinases can also regulate ETS1 phosphorylation, although these findings have been reported primarily in tumors [53, 54]. Whether similar kinase-mediated regulation occurs in the kidney, regarding Klotho-dependent mitochondrial modulation, remains to be determined. Regarding the regulation of PKCα expression by Klotho, we propose, based on existing literature and our previous research, that Klotho likely modulates PKCα expression by influencing calcium homeostasis [25, 55]. It should also be noted that the STZ/HFD model used herein, while suitable for studying early DKD events, does not fully recapitulate the advanced pathological stages of human disease.

## Conclusion

In summary, our findings delineate a novel Klotho/PKCα/ETS1/CYB5R4 signaling axis in PTECs. By modulating PKCα-mediated ETS1 activation, Klotho restores CYB5R4-dependent mitochondrial function, thereby attenuating the progression of DKD. This mechanistic insight not only enhances our understanding of DKD pathogenesis but also highlights potential therapeutic targets for preserving mitochondrial health and renal function in patients with diabetes. Targeting this signaling axis may provide a promising strategy for preventing or delaying DKD by concurrently addressing mitochondrial dysfunction, oxidative stress, and transcriptional dysregulation in PTECs.

## Supplementary Information

Supplementary Material 1. Figure S1 Histopathological changes in renal biopsies at progressive DKD stages and various weeklyintervals of DM. A-C) Renal histology of kidney sections from healthy controls and DKD (II-IV) patients was evaluated using HE and PASstaining, followed by semiquantitative analysis of morphological changes, along with immunofluorescence for NGAL and AQP1 expression.D) Schematic timeline of the diabetic mouse model generation and kidney tissue collection. E) Longitudinal measurement of urinary NGAL inmouse groups over weeks (n=6 per group). F) Representative HE, PAS, and immunofluorescence images of diabetic mouse kidneys atdifferent weeks post-modeling, showing progressive glomerular and tubular alterations and a gradual reduction of the proximal tubular markerLTL, while SGLT2 expression was increased. G-H) Representative IHC images showing NGAL and Synapotopodin (Synpo) expression inkidney sections from the indicated groups. Data are expressed as mean ± SD. Statistical significance is indicated as ns, no significant, **P<0.01, ***P< 0.001.

Supplementary Material 2. Figure S2 The protective role of Klotho in DKD progression through the maintenance of PTEC function. A) IF staining showing Klotho with AQP1 in mouse groups over weeks. B) Longitudinal measurement of urinary NGAL in mouse groups over weeks (n=6 per group). C, D) Representative images of renal histology in mouse groups over weeks, showing morphological changes in glomeruli and tubules by HE and PAS staining. E) Representative IHC images showing Synpo expression in kidney sections from the indicated groups. F) Representative TEM images showing foot process from the indicated groups. Data are expressed as mean ± SD. Statistical significance is indicated as ns, no significant, *P < 0.05, **P < 0.01.

Supplementary Material 3. Figure S3 Mitochondrial protection by Klotho in PTECs. A) IF staining showing podocyte markers Nephrin, WT1 in mouse groups over weeks. B) Single-cell UMAP plot with color annotations for cell type and cell distribution across groups. C) Representative images of cell mitochondria by TEM and Mitotracker, treated with Klotho. D, E) Western blot analysis of Cleaved-Caspase3, Bax, Cyt C, COXIV, VDAC1, TOM20 between HK-2 cells treated as HG and Klotho, and the transport of Cyt C into the cytoplasm (n=3). F-H) Assessment of cellular ATP content in HK-2 cells using a luminescence-based assay, mitochondrial TOM20 morphology, mitochondrial superoxide levels with MitoSOX, and mitochondrial membrane potential with TMRE and JC-1 in HK-2 cells treated with HG and Klotho. Data are expressed as mean ± SD. Statistical significance is indicated as *P < 0.05, **P < 0.01, ***P < 0.001. Abbreviations: PT, proximal tubule; CD, collecting duct; DCT, distal convoluted tubule; LOH, loop of Henle; EC, endothelial cells; T, T cell; B, B cell; Fib, fibroblast; MP, macrophage.

Supplementary Material 4. Figure S4 CYB5R4 is a key mediator of Klotho-induced recovery of mitochondrial function. A-H) Evaluation of CYB5R4 expression, Cyt C distribution in mitochondrial and cytosolic fractions, mitochondrial morphology (TOM20), mitochondrial superoxide levels (MitoSOX), and cellular ATP content in HK-2 cells with CYB5R4 knockdown or overexpression. I) Assessment of mitochondrial membrane potential by JC-1 staining in HK-2 cells with CYB5R4 knockdown or overexpression. J) Immunofluorescence staining showing CYB5R4 expression in HK-2 cells treated with high glucose (HG) or Klotho. Data are expressed as mean ± SD. Statistical significance is indicated as *P < 0.05, **P < 0.01.

Supplementary Material 5. Figure S5 CYB5R4 is a critical mediator of Klotho's renoprotection against DKD. A) Schematic diagram of the experimental design for AAV-mediated renal in situ injection in mice. B, C) Representative images of renal histology in mouse groups over weeks by HE and PAS staining, as well as tubule mitochondria by TEM.

Supplementary Material 6. Figure S6 Transcriptional suppression of CYB5R4 by ETS1. A, B) Validation of non-biotinylated and biotinylated CYB5R4 promoter probes by electrophoresis and Sanger sequencing for DNA pull-down assay, and identification of ETS1 as a CYB5R4 promoter–binding protein by mass spectrometry. C, D) Assessment of HK-2 cell CYB5R4 expression and mitochondrial membrane potential by JC-1 in ETS1 inhibition or overexpression. E) IF staining showing localization of ETS1 with COXIV between HK-2 cells treated as HG and Klotho. F) Western blot analysis of ETS1. G, H) IF staining showing pETS1 with COXIV in DKD (II-IV) patients and mouse groups at 8 weeks post-model induction. Data are expressed as mean ± SD. Statistical significance is indicated as ***P < 0.001.

Supplementary Material 7. Figure S7 PKCα downregulation by Klotho in the context of DKD. A, B) IF and IHC staining showing PKC with AQP1 between HC and DKD patients. C-F) IF and IHC staining showing PKC with AQP1 at 8 weeks in mouse groups. G) IHC staining showing classical PKC isoforms (α, β, and γ) at 8 weeks in Klotho inhibition or overexpression. H) IHC staining showing PKCα between HC and DKD patients.

Supplementary Material 8. Figure S8 PKCα phosphorylates and activates ETS1, which transcriptionally represses CYB5R4 expression. A) Immunofluorescence analysis of PKCα expression and its interference under high glucose conditions, and the effect of PKCα overexpression on CYB5R4, combined with transmission electron microscopy to assess mitochondrial changes in HK-2 cells. B) Mitochondrial morphology (TOM20), structure (Mitotracker), membrane potential (TMRE), and superoxide levels (MitoSOX) in HK-2 cells with PKCα inhibition or overexpression, assessed by immunofluorescence. C) Co-IP analysis showing the physical interaction between PKCα and ETS1 in PKCα-overexpressing or vector control cells. D-G) Western blot analysis of ETS1 subcellular localization in indicated group. Data are expressed as mean ± SD. Statistical significance is indicated as *P < 0.05, **P < 0.01, ***P < 0.001.

Supplementary Material 9. Supplementary Materials and Methods.

## Data Availability

All data and materials used in this study are available from the corresponding author upon reasonable request.
