# Supplementary material for: Modulation of PKCα/ETS1 by klotho restores CYB5R4-dependent mitochondrial function in proximal tubular epithelial cells to attenuate the progression of diabetic kidney disease
Source: Cardiovasc Diabetol. 2026 Mar 28;25:143. doi: 10.1186/s12933-026-03150-y (PMC13151253; doi:10.1186/s12933-026-03150-y)

Figure S1. Histopathological changes in renal biopsies at progressive DKD stages and various weekly intervals of DM.

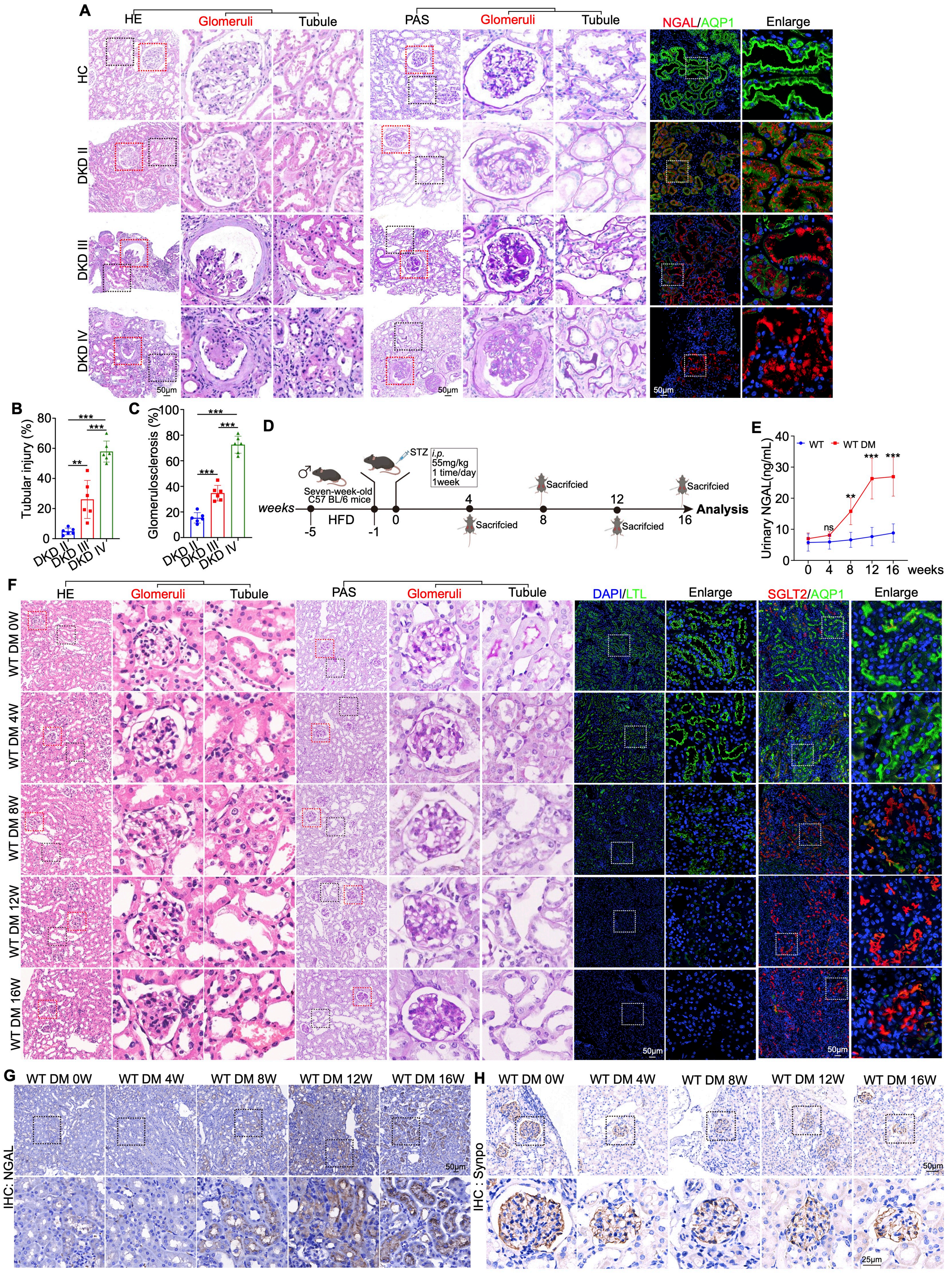

Supplement: Supplementary file 1 — Supplementary Material 1. Figure S1 Histopathological changes in renal biopsies at progressive DKD stages and various weeklyintervals of DM. A-C) Renal histology of kidney sections from healthy controls and DKD (II-IV) patients was evaluated using HE and PASstaining, followed by semiquantitative analysis of morphological changes, along with immunofluorescence for NGAL and AQP1 expression.D) Schematic timeline of the diabetic mouse model generation and kidney tissue collection. E) Longitudinal measurement of urinary NGAL inmouse groups over weeks (n=6 per group). F) Representative HE, PAS, and immunofluorescence images of diabetic mouse kidneys atdifferent weeks post-modeling, showing progressive glomerular and tubular alterations and a gradual reduction of the proximal tubular markerLTL, while SGLT2 expression was increased. G-H) Representative IHC images showing NGAL and Synapotopodin (Synpo) expression inkidney sections from the indicated groups. Data are expressed as mean ± SD. Statistical significance is indicated as ns, no significant, **P<0.01, ***P< 0.001. [file 12933_2026_3150_MOESM1_ESM.pdf]
