# Supplementary figures and images for: Modulation of PKCα/ETS1 by klotho restores CYB5R4-dependent mitochondrial function in proximal tubular epithelial cells to attenuate the progression of diabetic kidney disease
Source: Cardiovasc Diabetol. 2026 Mar 28;25:143. doi: 10.1186/s12933-026-03150-y (PMC13151253; doi:10.1186/s12933-026-03150-y)

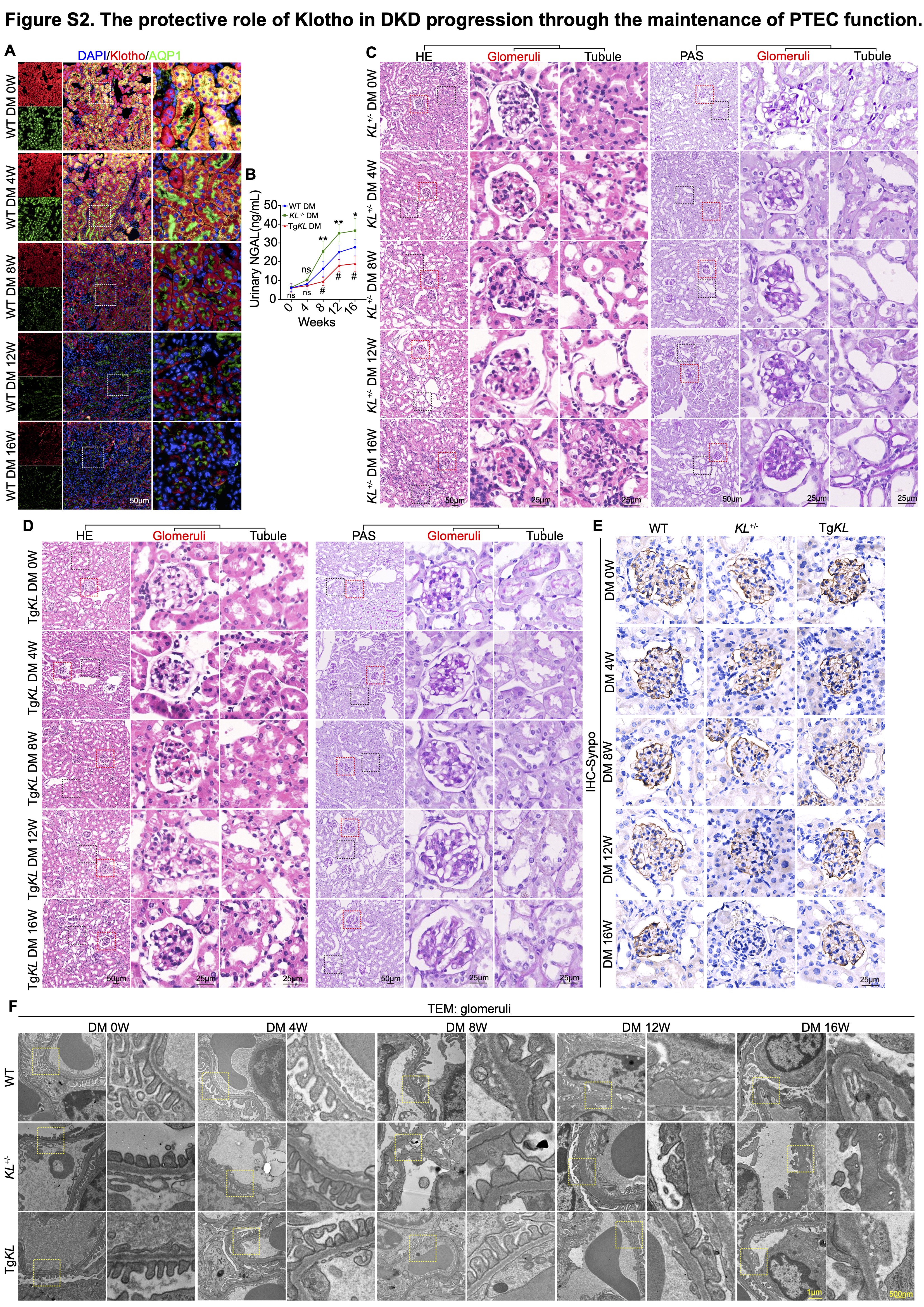

Supplement: Supplementary file 2 — Supplementary Material 2. Figure S2 The protective role of Klotho in DKD progression through the maintenance of PTEC function. A) IF staining showing Klotho with AQP1 in mouse groups over weeks. B) Longitudinal measurement of urinary NGAL in mouse groups over weeks (n=6 per group). C, D) Representative images of renal histology in mouse groups over weeks, showing morphological changes in glomeruli and tubules by HE and PAS staining. E) Representative IHC images showing Synpo expression in kidney sections from the indicated groups. F) Representative TEM images showing foot process from the indicated groups. Data are expressed as mean ± SD. Statistical significance is indicated as ns, no significant, *P < 0.05, **P < 0.01. [file 12933_2026_3150_MOESM2_ESM.jpg]

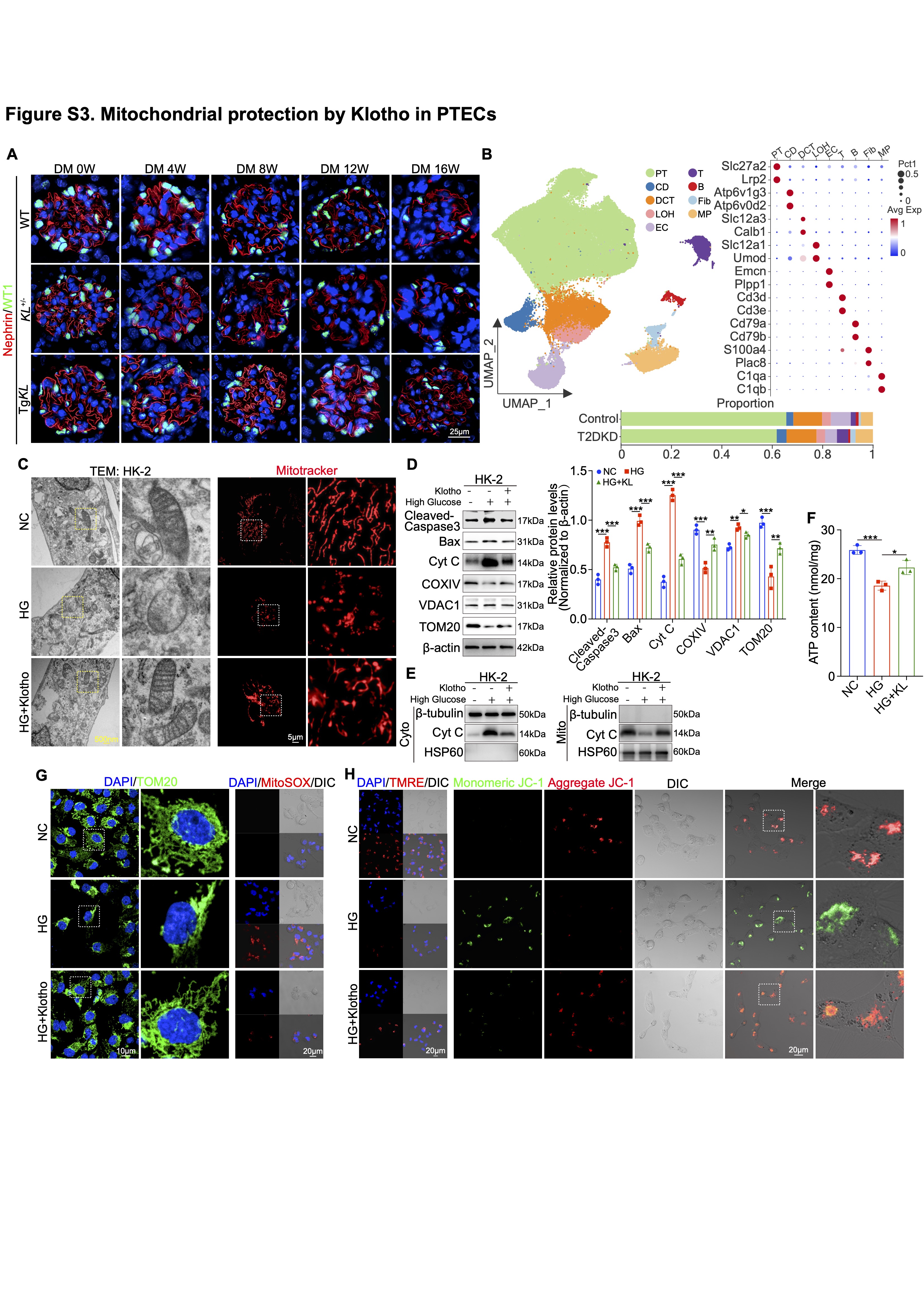

Supplement: Supplementary file 3 — Supplementary Material 3. Figure S3 Mitochondrial protection by Klotho in PTECs. A) IF staining showing podocyte markers Nephrin, WT1 in mouse groups over weeks. B) Single-cell UMAP plot with color annotations for cell type and cell distribution across groups. C) Representative images of cell mitochondria by TEM and Mitotracker, treated with Klotho. D, E) Western blot analysis of Cleaved-Caspase3, Bax, Cyt C, COXIV, VDAC1, TOM20 between HK-2 cells treated as HG and Klotho, and the transport of Cyt C into the cytoplasm (n=3). F-H) Assessment of cellular ATP content in HK-2 cells using a luminescence-based assay, mitochondrial TOM20 morphology, mitochondrial superoxide levels with MitoSOX, and mitochondrial membrane potential with TMRE and JC-1 in HK-2 cells treated with HG and Klotho. Data are expressed as mean ± SD. Statistical significance is indicated as *P < 0.05, **P < 0.01, ***P < 0.001. Abbreviations: PT, proximal tubule; CD, collecting duct; DCT, distal convoluted tubule; LOH, loop of Henle; EC, endothelial cells; T, T cell; B, B cell; Fib, fibroblast; MP, macrophage. [file 12933_2026_3150_MOESM3_ESM.jpg]

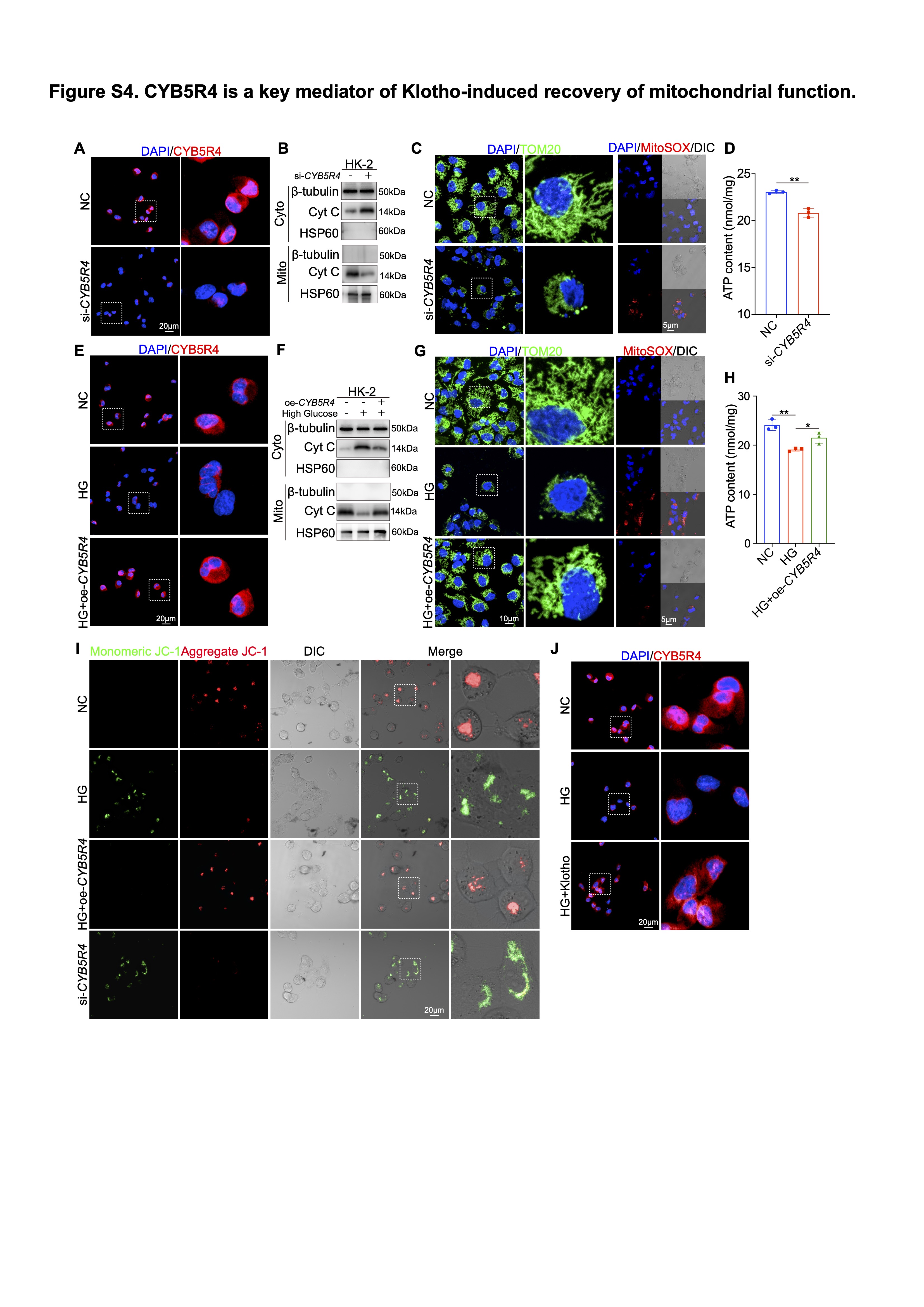

Supplement: Supplementary file 4 — Supplementary Material 4. Figure S4 CYB5R4 is a key mediator of Klotho-induced recovery of mitochondrial function. A-H) Evaluation of CYB5R4 expression, Cyt C distribution in mitochondrial and cytosolic fractions, mitochondrial morphology (TOM20), mitochondrial superoxide levels (MitoSOX), and cellular ATP content in HK-2 cells with CYB5R4 knockdown or overexpression. I) Assessment of mitochondrial membrane potential by JC-1 staining in HK-2 cells with CYB5R4 knockdown or overexpression. J) Immunofluorescence staining showing CYB5R4 expression in HK-2 cells treated with high glucose (HG) or Klotho. Data are expressed as mean ± SD. Statistical significance is indicated as *P < 0.05, **P < 0.01. [file 12933_2026_3150_MOESM4_ESM.jpg]

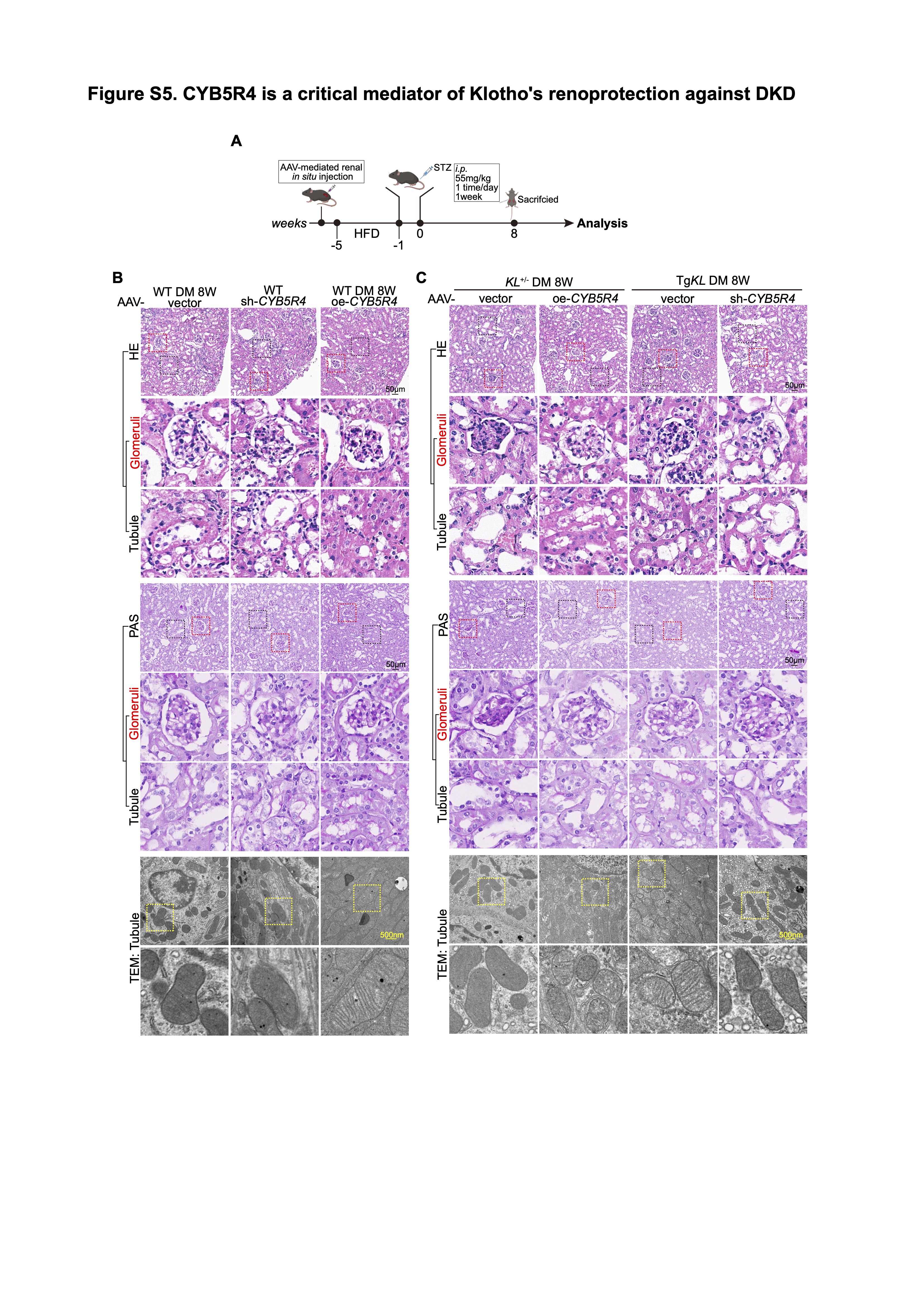

Supplement: Supplementary file 5 — Supplementary Material 5. Figure S5 CYB5R4 is a critical mediator of Klotho's renoprotection against DKD. A) Schematic diagram of the experimental design for AAV-mediated renal in situ injection in mice. B, C) Representative images of renal histology in mouse groups over weeks by HE and PAS staining, as well as tubule mitochondria by TEM. [file 12933_2026_3150_MOESM5_ESM.jpg]

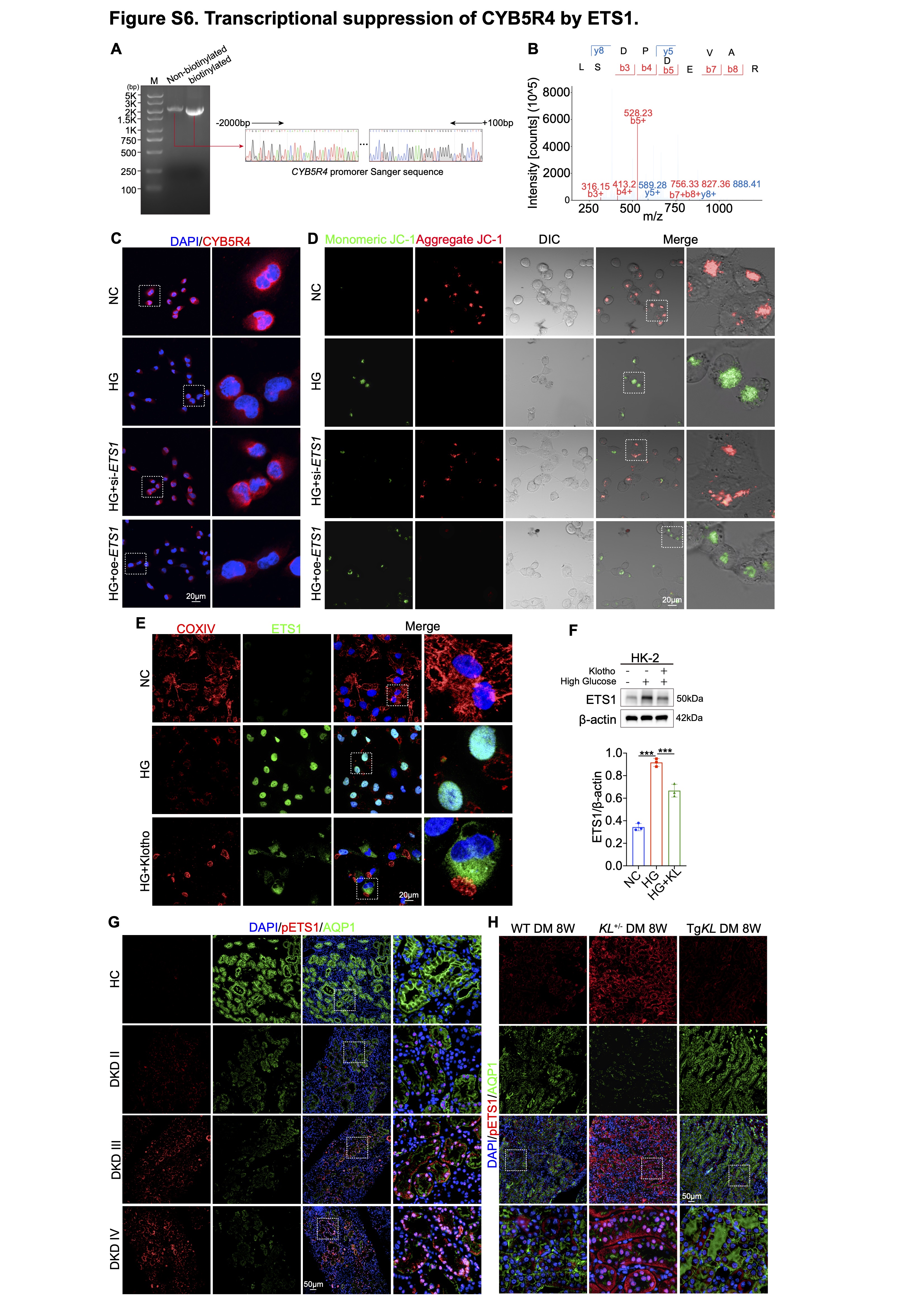

Supplement: Supplementary file 6 — Supplementary Material 6. Figure S6 Transcriptional suppression of CYB5R4 by ETS1. A, B) Validation of non-biotinylated and biotinylated CYB5R4 promoter probes by electrophoresis and Sanger sequencing for DNA pull-down assay, and identification of ETS1 as a CYB5R4 promoter–binding protein by mass spectrometry. C, D) Assessment of HK-2 cell CYB5R4 expression and mitochondrial membrane potential by JC-1 in ETS1 inhibition or overexpression. E) IF staining showing localization of ETS1 with COXIV between HK-2 cells treated as HG and Klotho. F) Western blot analysis of ETS1. G, H) IF staining showing pETS1 with COXIV in DKD (II-IV) patients and mouse groups at 8 weeks post-model induction. Data are expressed as mean ± SD. Statistical significance is indicated as ***P < 0.001. [file 12933_2026_3150_MOESM6_ESM.jpg]

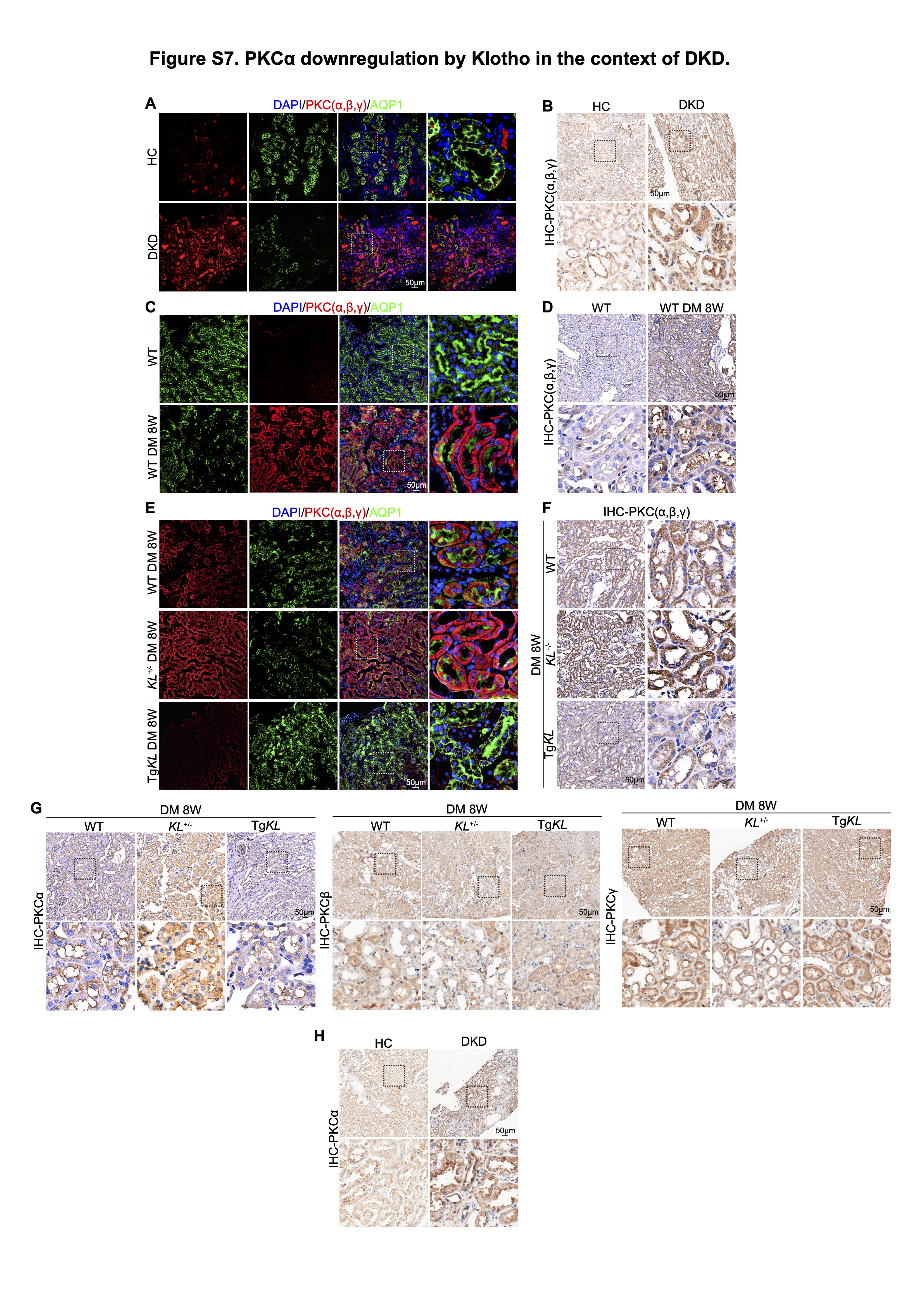

Supplement: Supplementary file 7 — Supplementary Material 7. Figure S7 PKCα downregulation by Klotho in the context of DKD. A, B) IF and IHC staining showing PKC with AQP1 between HC and DKD patients. C-F) IF and IHC staining showing PKC with AQP1 at 8 weeks in mouse groups. G) IHC staining showing classical PKC isoforms (α, β, and γ) at 8 weeks in Klotho inhibition or overexpression. H) IHC staining showing PKCα between HC and DKD patients. [file 12933_2026_3150_MOESM7_ESM.jpg]

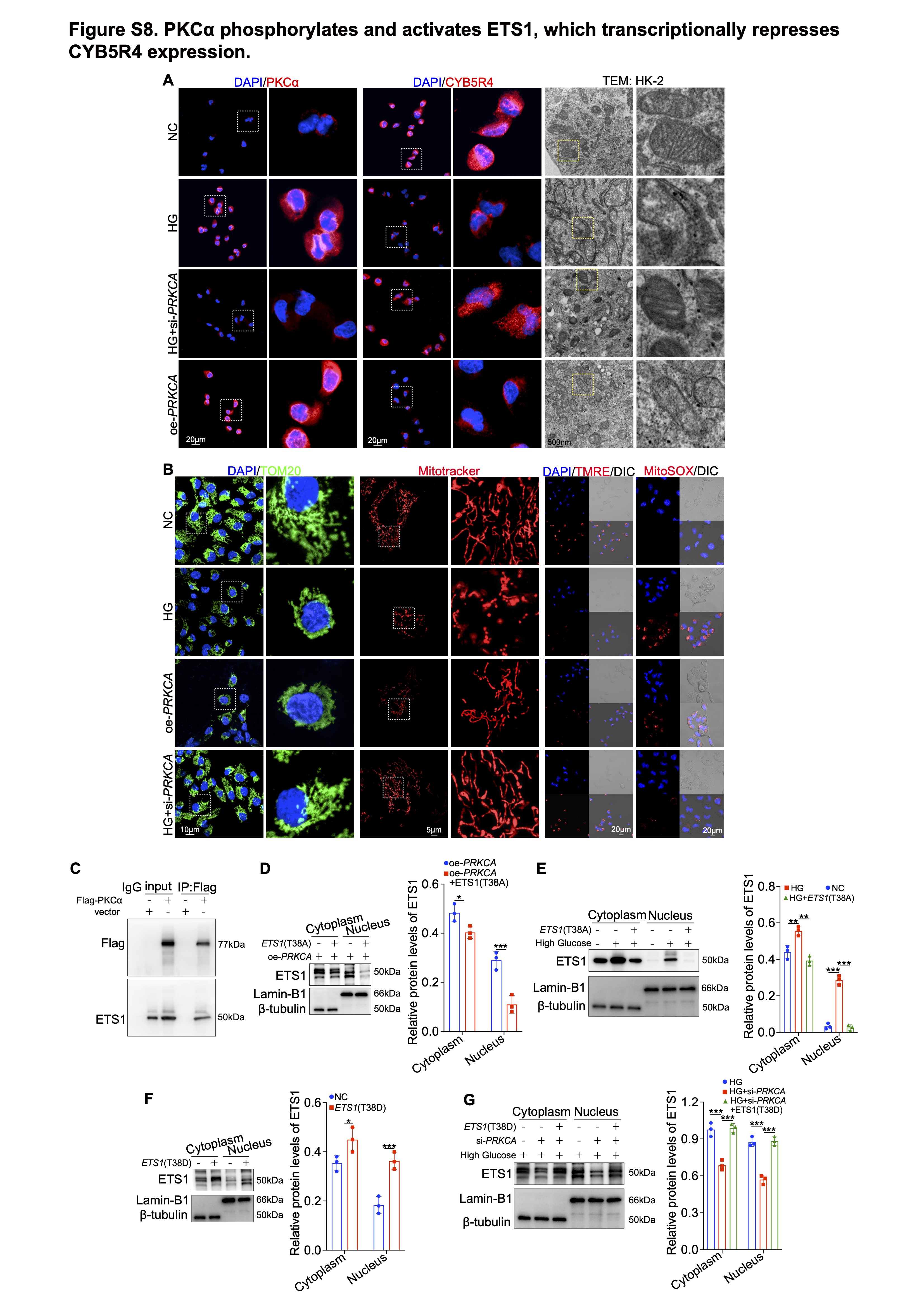

Supplement: Supplementary file 8 — Supplementary Material 8. Figure S8 PKCα phosphorylates and activates ETS1, which transcriptionally represses CYB5R4 expression. A) Immunofluorescence analysis of PKCα expression and its interference under high glucose conditions, and the effect of PKCα overexpression on CYB5R4, combined with transmission electron microscopy to assess mitochondrial changes in HK-2 cells. B) Mitochondrial morphology (TOM20), structure (Mitotracker), membrane potential (TMRE), and superoxide levels (MitoSOX) in HK-2 cells with PKCα inhibition or overexpression, assessed by immunofluorescence. C) Co-IP analysis showing the physical interaction between PKCα and ETS1 in PKCα-overexpressing or vector control cells. D-G) Western blot analysis of ETS1 subcellular localization in indicated group. Data are expressed as mean ± SD. Statistical significance is indicated as *P < 0.05, **P < 0.01, ***P < 0.001. [file 12933_2026_3150_MOESM8_ESM.jpg]
