# Supplementary material for: Modulation of PKCα/ETS1 by klotho restores CYB5R4-dependent mitochondrial function in proximal tubular epithelial cells to attenuate the progression of diabetic kidney disease
Source: Cardiovasc Diabetol. 2026 Mar 28;25:143. doi: 10.1186/s12933-026-03150-y (PMC13151253; doi:10.1186/s12933-026-03150-y)
Supplement: Supplementary file 9 — Supplementary Material 9. Supplementary Materials and Methods. [file 12933_2026_3150_MOESM9_ESM.docx]

**Supplementary Materials and methods**

***Cell Culture and Treatments*:** Human Kidney-2 (HK-2, TCH-C400, RRID: CVCL_0302) cells were purchased from Haixing Biosciences and cultured in HK-2-specific medium (TCH-G400, Haixing Biosciences). Cells were authenticated by short tandem repeat (STR) profiling and routinely monitored for bacterial, fungal, and mycoplasma contamination. Only contamination-free cells were used in experimental analyses. Prior to treatment, cells were pre-incubated with recombinant human Klotho protein (500 pM, ab84072, Abcam) for 2 hours. For experimental conditions, cells were exposed to 30 mM high glucose (HG) for 48 hours. To investigate the effects of PRKCA, ETS1, and CYB5R4, overexpression was achieved using plasmids (YouBio). Knockdown of PRKCA, ETS1 and CYB5R4 was performed using small interfering RNAs (siRNAs), which were synthesized by Geecreate (Shanghai, China). Plasmids or siRNAs were transfected into HK-2 cells using Lipofectamine 3000 (L3000015, Thermo Fisher Scientific) following the manufacturer’s protocol. Briefly, cells seeded in 6-well plates were transfected at 60-70% confluence by incubating with transfection complexes prepared in Opti-MEM (31985070, Thermo Fisher Scientific) for 10 minutes. After 6–8 hours, the medium was replaced, and cells were harvested 24-48 hours post-transfection for subsequent analysis.

***Kidney Histology*:** Kidney tissues were fixed in 4% paraformaldehyde (G1101, Servicebio), embedded in paraffin, and sectioned into 3.5 µm thick slices. Tissue slides were stained with hematoxylin-eosin (HE) and periodic acid-Schiff (PAS) according to the manufacturer's instructions (G1120, G1281, Solarbio). Stained sections were then examined using a PanoCube microscope (Meca Scientific).

***Immunohistochemistry (IHC) and Immunofluorescence (IF)*:** Kidney tissue slides were dewaxed, rehydrated, and subjected to heat-induced antigen retrieval using EDTA (ZLI-9072, ZSGB-BIO). For IHC staining, slides were processed with the enhanced polymer test kit (PV9001, ZSGB-BIO), incubated with primary antibodies for 1 hour at room temperature, and developed using a DAB substrate kit (DA1010, Solarbio). For IF staining, cells were washed with PBS, fixed with 4% paraformaldehyde for 10 minutes at room temperature, and blocked with 10% goat serum (ZLI-9021, ZSGB-BIO). Slides and cells were then incubated with primary antibodies overnight at 4°C. Afterward, they were incubated with the following secondary antibodies for 1 hour at room temperature: goat anti-Rabbit IgG Alexa Fluor^TM^ 555 (1:800, A-21428, Invitrogen), goat anti-Mouse IgG Alexa Fluor^TM^ 647 (1:800, A-21235, Invitrogen), and goat anti-Guinea Pig IgG Alexa Fluor^TM^ 568 (1:800, A-11075, Invitrogen). Nuclei were counterstained with DAPI (1:1000, C1002, Beyotime) for 10 minutes at room temperature, and slides were mounted with an antifade mounting medium (P0126, Beyotime). Images were captured using a PanoCube (Meca Scientific). Primary antibodies used: Anti-Nephrin antibody (1:50, GP-N2, PROGEN), Anti-Wilms Tumor Protein antibody (WT1, 1:50, ab89901, Abcam), Anti-Synaptopodin antibody (Synpo, 1:2000, 21064-1-AP, Proteintech), Anti-AQP1 antibody (1:50, sc-55466, Santa Cruz), Anti-Calbindin D28K antibody (1:400, 214004, Synaptic Systems), Anti-AQP2 antibody (1:200, 29386-1-AP, Proteintech), Anti-Bax antibody (1:100, 380709, ZENBIO), Anti-Caspase 3 (Cleaved Asp175) antibody (1:50, PA5-114687, Invitrogen), Anti-Klotho antibody (1:100, PA5-88303, Invitrogen) for human kidney tissue, Anti-Klotho antibody (1:100, 28100-1-AP, Proteintech) for mouse kidney tissue, Anti-Lipocalin-2 / NGAL antibody [EPR21092] (1:2000, ab216462, Abcam), Anti-COXIV antibody (1:200, AWA10134, Abiowell), Anti-TOM20 antibody (1:200, AWA10405, Abiowell), Lotus Tetragonolobus Lectin (LTL), Fluorescein (1:200, FL-1321, VectorLabs), Anti-SGLT2 Polyclonal antibody (1:200, 24654-1-AP, Proteintech), Anti-CYB5R4 antibody (1:100, PA5-103468, Invitrogen) for tissue staining, Anti-CYB5R4 antibody (1:200, AWA50665, Abiowell) for cell staining, Anti-ETS1 antibody (1:100, ab307672, Abcam), Anti-phospho-ETS1 (Thr38) antibody (1:50, 310267, ZENBIO), Anti-PKC alpha + beta 2 + gamma antibody (1:400, ab184746, Abcam), Anti-PKC alpha antibody (PKCα, 1:200, A11107, ABclonal), Anti-PKC beta antibody (PKCβ, 1:400, A21241, ABclonal), Anti-PKC gamma antibody (PKCγ, 1:200, 29208-1-AP, Proteintech). Due to reported specificity limitations of several commercially available anti-SGLT2 antibodies in human kidney tissue, as demonstrated by knockout-based validation studies ((Hirose *et al*. Reliable Detection of SGLT2 Protein by Knockout-Based Antibody Characterization. *Hypertension,* 2025), human SGLT2 immunostaining results were not included in the final analysis to avoid potential misinterpretation.

***Western blot*:** Total protein from kidney tissue and cells was extracted using RIPA lysis buffer (WB3100, NCM Biotech), supplemented with 1% protease inhibitor cocktail (C0001, TargetMol) and 1% phosphatase inhibitor cocktail (C0004, TargetMol). For subcellular localization of ETS1, protein was isolated using the nuclear and cytoplasmic extraction kit (E101, Vazyme). Protein concentration was determined using the detergent-compatible Bradford protein quantification kit (E211, Vazyme). Proteins were resolved by 7.5% or 12.5% SDS-PAGE, transferred to PVDF membranes, and blocked with 5% skim milk in TBST for 1 hour. Membranes were incubated with primary antibodies overnight at 4°C, followed by incubation with HRP-conjugated secondary antibodies (1:10,000, ZB-5301, ZB-2305, ZSGB-BIO). Protein bands were visualized using an ECL substrate (34580, ThermoFisher Scientific), and signal intensities were quantified using ImageJ software. Primary antibodies used: Anti-AQP1 antibody (1:5000, 20333-1-AP, Proteintech), Anti-AQP2 antibody (1:1000, 29386-1-AP, Proteintech), Anti-Calbindin D28K antibody (1:15,000, 14479-1-AP, Proteintech), Anti-Wilms Tumor Protein antibody (WT1, 1:500, ET1610-45, HUABIO), Anti-NPHS2 antibody (1:500, ET7107-34, HUABIO), Anti-active Caspase-3 antibody (1:500, A11021, Abclonal), Anti-Bax antibody (1:1000, 380709, ZENBIO), Anti-PINK1 antibody (1:2000, 23274-1-AP, Proteintech), Anti-Parkin antibody (1:1000, YT3593, Immunoway), Anti-VDAC1 antibody (1:5000, 81538-1-RR, Proteintech), Anti-COXIV antibody (1:1000, AWA10134, Abiowell), Anti-Cytochrome c antibody (1:4000, 10993-1-AP, Proteintech), Anti-HSP60 antibody (1:50000, ET1609-45, HUABIO), Anti-TOM20 antibody (1:1000, AWA10405, Abiowell), Anti-CYB5R4 antibody (1:1000, AWA50665, Abiowell) for cell lysate, Anti-CYB5R4 antibody (1:1000, 12626-1-AP, Proteintech) for mouse kidney lysate, Anti-ETS1 antibody (1:5000, ET1705-23, HUABIO), Anti-PKC alpha + beta 2 + gamma antibody (1:1000, ab184746, Abcam), Anti-PKC alpha antibody (PKCα, 1:1000, A11107, ABclonal), Anti-PKC beta antibody (PKCβ, 1:4000, A21241, ABclonal), Anti-PKC gamma antibody (PKCγ, 1:2000, 29208-1-AP, Proteintech), Anti-phospho-ETS1 (Thr38) antibody (1:500, 310267, ZENBIO), Anti-β-actin antibody (1:5000, AWA80113, Abiowell), Anti-β-tubulin antibody (1:1000, AWA80025, Abiowell), Anti-Lamin B1 antibody (1:1000, F0523, Selleck).

***Transmission Electron Microscopy (TEM)*:** Kidney tissue pieces and cultured HK-2 pelleted cells were fixed in 4% glutaraldehyde fixative solution (P1127, Servicebio). Ultrastructural analysis was performed using a Transmission Electron Microscope (JEOL JEM-1400Plus) at the Department of Electron Microscopy, Chongqing Medical University.

***Analysis of Single-Cell RNA-Seq Data*:** Our study utilized transcriptome data from GSE261356 for a comprehensive analysis. A computational pipeline integrating data integration, clustering, and annotation was implemented using the CytoNavigator platform (https://sc.novelbrain.com/). Briefly, cells were filtered based on the following criteria: >200 genes and <50% mitochondrial UMIs. Data normalization was performed using Seurat (version 4.1.1, https://satijalab.org/seurat/). Scaled data underwent principal component analysis (PCA) using 2000 variable genes, with the top 10 principal components used for t-SNE/UMAP dimensionality reduction. Unsupervised clustering and marker detection (Wilcoxon, log₂FC > 0.25, *p*<0.05) were applied, followed by subclustering for more refined annotation. Gene Ontology (GO) analysis of differentially expressed genes (DEGs) and marker genes was performed using annotations from NCBI, Gene Ontology, and UniProt databases. Significant GO terms were identified using Fisher’s exact test with p-values. Visualizations were generated using OECloud tools (https://cloud.oebiotech.com).

***Assessment of Mitochondrial Morphology and Function*:** Mitochondria were labeled using MitoTracker Red (40741ES50, Yeasen). Cells were incubated with 200 nM MitoTracker Red in culture medium at 37°C for 30 minutes. Imaging was conducted using a Polar-SIM system (Airy Technologies). Mitochondrial membrane potential was assessed using the fluorescent dye TMRE (T19072, TargetMol) at a concentration of 5 μM, with cells incubated for 20 minutes at 37°C. Additionally, changes in mitochondrial membrane potential were evaluated using the JC-1 fluorescent probe (40706ES60, Yeasen) according to the manufacturer’s protocol. Mitochondrial superoxide levels were assessed using MitoSOX Red (M36008, Invitrogen). Cells were incubated with 500 nM MitoSOX Red at 37°C for 20 minutes, following the manufacturer's instructions. Confocal microscopy (Nikon AX) was used for imaging.

***LC-MS/MS Proteomics Analysis*:** The experiment was conducted as follows: HK-2 cells were cultured and divided into three experimental groups (Normal Glucose (NC). High Glucose (HG). High Glucose + Klotho (HG+KL)). Following treatment, cells were digested with trypsin and centrifuged. The resulting cell pellets were washed four times with ice-cold PBS, then transported on dry ice. Protein expression changes were analyzed using 4D-FastDIA-based quantitative proteomics. Functional classification analysis of differentially expressed proteins was conducted using the Clusters of Orthologous Groups (COG) database, with eukaryotic protein orthologs categorized under the term KOG.

***Biotin-Streptavidin Affinity Pull-Down and LC-MS/MS Integration*:** To investigate CYB5R4 gene promoter-protein interactions, a biotin-streptavidin-mediated pull-down assay was performed using amplified DNA (Forward primer: 5’-AATGACAGTATTTAATTCTCAAGACTGAAT-3’; Reverse primer: 5’-CAGCATCTTCAAACCCCGG-3’) with a 5’-biotin modification on the sense strand. The reaction mixture, consisting of 5 µg of biotinylated oligonucleotide, 500 µg of nuclear protein extract, and Mag Beads Streptavidin (C0090, TargetMol), was incubated overnight at 4°C. Non-biotinylated DNA served as the control. After incubation, the beads were washed stringently with PBS and subjected to LC-MS/MS to validate candidate transcription factors (TFs). The identified TFs were cross-referenced with JASPAR (v2024) and analyzed using WikiPathways (https://www.wikipathways.org/) to identify key hub transcription factors.

***CUT&Tag-qPCR Assay*:** CUT&Tag was performed using the TD904 Kit (Vazyme) following the manufacturer’s instructions. Briefly, cells were collected, washed, and bound to pre-activated ConA Beads Pro, then incubated overnight at 4℃ with ETS1 antibody (14069, CST) or Rabbit IgG control (A7016, Beyotime) in Antibody Buffer. After washing with Dig-Wash Buffer, samples were incubated with secondary antibody (Ab207, Vazyme) for 60 min at room temperature, followed by incubation with pA/G‑Tnp Pro transposase complexes for 1 h. Tagmentation was carried out in 1× TTBL at 37℃ for 60 min and stopped with 10 % SDS and DNA Spike-in. DNA fragments were extracted using DNA Extract Beads Pro, washed with 1× B&W Buffer, and eluted in ddH_2_O. For qPCR, extracted DNA was heated with Stop Buffer (TD904-C1) at 95℃ for 5 min, and the supernatant was used as template with SYBR Green Master Mix (Q712, Vazyme) and primers CYB5R4-Forward (5’‑AGTAGTGGCTGTTGACATTTG‑3’) and CYB5R4-Reverse (5’‑AAGCA GTACCATCACACTACTT‑3’). Relative enrichment was calculated using the 2^(-ΔΔCt) method with the IgG control as reference.

***RNA Isolation, cDNA Synthesis, and Quantitative PCR (qPCR)*:** Total RNA was extracted from cells using the SteadyPure Quick RNA Extraction Kit (AG21023, Accurate Biology) according to the manufacturer's protocol, and RNA concentration and purity were assessed. First-strand cDNA was synthesized from 1 µg of RNA using the HiScript IV 1st Strand cDNA Synthesis Kit (R412, Vazyme). Quantitative PCR (qPCR) was performed using the Taq Pro Universal SYBR qPCR Master Mix (Q712, Vazyme) with specific primers, and gene expression was analyzed using the 2^(-ΔΔCt) method. The primer sequences for amplification were as follows: CYB5R4 Forward: 5’‑GGGGGCGTAGCAAGGTA‑3’, CYB5R4 Reverse: 5’‑GGCTGACATTATAAACGAAACCT‑ 3’, β-actin Forward: 5’‑AATCTGGCACCACACCTTCTACAA‑3’, and β-actin Reverse: 5’‑GGAT AGCACAGCCTGGATAGCAA‑3’.
